# An epigenetic pathway in rice connects genetic variation to anaerobic germination and seedling establishment

**DOI:** 10.1093/plphys/kiab100

**Published:** 2021-02-26

**Authors:** Lina Castano-Duque, Sharmistha Ghosal, Fergie A Quilloy, Thomas Mitchell-Olds, Shalabh Dixit

**Affiliations:** 1 Department of Biology, Duke University, Durham, NC 27708, USA; 2 Rice Breeding Platform, International Rice Research Institute. Pili Drive, Los Baños, Laguna 4031, Philippines

## Abstract

Rice production is shifting from transplanting seedlings to direct sowing of seeds. Following heavy rains, directly sown seeds may need to germinate under anaerobic environments, but most rice (*Oryza sativa*) genotypes cannot survive these conditions. To identify the genetic architecture of complex traits, we quantified percentage anaerobic germination (AG) in 2,700 (wet-season) and 1,500 (dry-season) sequenced rice genotypes and performed genome-wide association studies (GWAS) using 693,502 single nucleotide polymorphisms. This was followed by post-GWAS analysis with a generalized SNP-to-gene set analysis, meta-analysis, and network analysis. We determined that percentage AG is intermediate-to-high among *indica* subpopulations, and AG is a polygenic trait associated with transcription factors linked to ethylene responses or genes involved in metabolic processes that are known to be associated with AG. Our post-GWAS analysis identified several genes involved in a wide variety of metabolic processes. We subsequently performed functional analysis focused on the small RNA and methylation pathways. We selected *CLASSY 1 (CLSY1)*, a gene involved in the RNA-directed DNA methylation (RdDm) pathway, for further analyses under AG and found several lines of evidence that *CLSY1* influences AG. We propose that the RdDm pathway plays a role in rice responses to water status during germination and seedling establishment developmental stages.

## Teaser

An RNA-directed DNA methylation factor functions in anaerobic germination and seedling growth in rice.

## Introduction

Rice (*Oryza sativa*) is a staple food for 50% of the global population and ensuring its climate resilience and yield stability is key to ensuring food security. Present-day rice cultivation is affected by a variety of climate-related and social challenges, such as water shortages and unavailability of labor in major rice-growing countries, which make transplanting in paddies difficult and cost-intensive (Kumar and Ladha, 2011). Thus, direct seeding of rice into dry soil is being developed as an alternate method of crop establishment. However, ensuring adequate germination of seeds under varying environmental conditions and management practices is key to success in this system. Rice seeds may need to germinate under anaerobic conditions due to flooding following rainstorms immediately after seeding (Ray et al., 2016). Successful anaerobic germination (AG) is an essential trait in rice cultivars developed for areas where flooding is common. Research is ongoing to find the quantitative trait locus (QTL) associated with AG tolerance, but little is known about natural variation for AG in rice. This knowledge gap calls for comprehensive genome-wide association studies (GWAS) and analysis of plant metabolic pathways to discover the genetic and biochemical architecture that controls AG (Miro and Ismail, 2013).

During flooding conditions, plants suffer from a shortage of energy, oxygen, and light, which leads to lower photosynthetic rates and yields (Voesenek and Bailey-Serres, 2013, 2015). Depending on the plant genotype, flood-tolerant plants are able to change their metabolism, root morphology, and anatomy during hypoxia (low-oxygen conditions; Voesenek and Bailey-Serres, 2013, 2015)*.* There are two flooding tolerance strategies known in domesticated rice(Voesenek and Bailey-Serres, 2013, 2015): low-oxygen escape syndrome (LOES) and low-oxygen quiescence syndrome (LOQS). LOES phenotypes have high energy consumption and are characterized by upward bending of the leaves, shoot elongation, pressurized flow of gas through porous tissues (Voesenek and Bailey-Serres, 2013, 2015), formation of aerenchyma (air spaces in plant tissue), root anatomical barriers that prevent oxygen loss, adventitious roots (Sauter, 2013), and gas films on leaves (Voesenek and Bailey-Serres, 2013, 2015). LOQS phenotypes tend to lower their metabolic rates, reducing cell division and overall growth in order to conserve energy for use when environmental conditions become favorable (Voesenek and Bailey-Serres, 2013, 2015).

LOES and LOQS phenotypes are strongly regulated by ethylene-responsive factor VII (ERF-VII) transcription activators (Voesenek and Bailey-Serres, 2013). During submergence, the LOES rice phenotype shows internode shoot elongation that leads to escape from underwater conditions as a result of ERF-VII SNORKEL (*SK1* and *SK2*; Hattori et al., 2009) alleles. In contrast, LOQS rice phenotypes display energy conservation by downregulating gene expression involving cell wall loosening and starch and sucrose catabolism, which lead to quiescence as a result of the ERF-VII *SUB1A-1* (Xu et al., 2006) allele (Voesenek and Bailey-Serres, 2013). These ERFs are controlled by low levels of oxygen and nitric oxide (NO) as well as ethylene signaling pathways that are key during low-oxygen conditions in LOES and LOQS (Voesenek and Bailey-Serres, 2015). Throughout the plant life cycle, there are several stages in which a plant can encounter flooding conditions. However, known genes that contribute to LOES and LOQS in juvenile plants have little influence when flooding occurs during seed germination (Ray et al., 2016).

Under AG, the plant must produce ATP at lower sucrose concentrations because flooded environments decrease the gas exchange that is required to produce sugars through photosynthesis (Ray et al., 2016). Starch reserves in the seed become key for AG because starch can be cleaved in several steps to produce pyruvate (Pompeiano et al., 2013; Kretzschmar et al., 2015), which is needed for ATP production. Also, in AG conditions, the plant switches from the tricarboxylic acid (Metcalf and Lampman, 1989) cycle to fermentative metabolism to produce ATP (Magneschi and Perata, 2009; Miro and Ismail, 2013; Ray et al., 2016). The seed senses elevated sucrose and low trehalose-6-phosphate (T6P), which leads to the production of alpha-amylase and breakdown of starch (Kretzschmar et al., 2015; Loreti et al., 2016). In rice, higher amylolytic activities have been positively correlated with coleoptile elongation 7 and 20 d after AG (Pompeiano et al., 2013; Pompeiano and Guglielminetti, 2016), and hypoxia leads to the breakdown of starch reserves through the starvation-inducible alpha-amylase enzyme, RA*my*3D. The induction of RA*my*3D is correlated with plant sensing of sugar content, which in AG-tolerant rice has been correlated with functional trehalose-6-P-phosphate phosphatase (OsTPP7) activity. *OsTPP7* contributes to a major QTL for AG tolerance in the Myanmar landrace Khao Hlan On (Kretzschmar et al., 2015). This enzyme converts T6P into trehalose and plays a crucial role in the modulation of local T6P/sucrose ratios. Further research on the AG trait may lead to the discovery of more genes and pathways that control AG in other rice genetic backgrounds.

Association analysis of whole-organism traits can incorporate evidence regarding selectively important functional variation (Daub et al., 2013, 2015). This can be combined with information on metabolic pathways and gene expression networks (Wang et al., 2010; Chan et al., 2011; Califano et al., 2012; Ramanan et al., 2012; Dembeck et al., 2015) to detect new pathways associated with a trait of interest. Such explicit pathway approaches in GWAS may detect enrichment of genes in a network even if individual associations do not attain genome-wide significance thresholds. For example, network-based analysis of olfactory behavior in *Drosophila melanogaster* showed enrichment for cell signaling and neural development genes associations that were not significant using whole-genome sequences without information on pathway context (Swarup et al., 2013). Similar approaches have been applied to metabolic pathways in maize (*Zea mays*; Li et al., 2013; Lipka et al., 2013; Owens et al., 2014), transcriptomic networks in Arabidopsis (Chan et al., 2011), and diseases in humans (Baranzini, 2013; Raj et al., 2013). Alternatively, genomic annotation information can dramatically reduce the number of candidate loci during fine-mapping (Pickrell, 2014; Rodgers-Melnick et al., 2015). Using approaches such as these, systems genetics can greatly improve the ability to find and understand the genes responsible for complex trait variation in plants.

Biological variability among rice genotypes in terms of successful AG, seedling establishment, and responses to variable water status involve complex genetic traits. Rice capacity to germinate under anaerobic conditions could involve several enzyme activities, including OsTPP7. Nevertheless, further screening for traits related to AG could lead to the discovery of more genes and pathways controlling AG (Miro and Ismail, 2013). In this study, we screened sequenced cultivars from 3,000 rice genome germplasm (Mansueto et al., 2016; Wang et al., 2018) from the International Rice Research Institute (IRRI) and determined that AG is a polygenic trait. We propose a novel stress response mechanism in rice that involves the RNA-directed DNA methylation (RdDm) pathway to control for germination and seedling establishment under flooding.

## Results

### *Indica* varieties have intermediate-to-high AG percentages

Rice percentage of germination (Eq. 1) was higher in control compared to flooded environment (Supplemental Figure S1) in both seasons. This indicated an overall reduction in germination under flooded conditions across rice populations. However, the extent of germination reduction at an individual level was dependent on the tolerance of the genotype. In order to capture this dependency, we integrated control and flooded phenotypic responses into a physiological metric called relative germination (Eq. 2). This trait allowed us to evaluate plant germination and seedling establishment under the assumption that high relative germination is indicative of a stable response to control and flooded environments, meaning that a similar level of germination happened in flooded and control environments. Relative germination accounted for plant response across water regimes and allowed the identification of germplasm with stable phenotype across environments. Heritability values for the wet season were 0.46 and 0.33 for control- and flooded-environment, respectively; and for the dry season were 0.9 and 0.96 for control- and flooded-environment, respectively. Over 50% of rice lines evaluated in the wet season (Supplemental Table S1) had relative germination equal to zero; consequently, the relative germination distribution was zero-inflated (Supplemental Figure S1). In the wet season, only 32% of the lines (895 out of 2,735 genotypes) had nonzero relative germination, whereas in the dry season, it was 91% (1,385 out of 1,509 genotypes). Despite having more plants capable of germinating in the dry season, the distribution of relative germination showed few rice lines with values above 0.5 (Supplemental Figure S1). Genotypes with relative germination higher than 0.5 were considered good *multi-environment germinators* because they germinated at a similar level in flooded and control environments. In the wet season, only 14% (129) of the germinated rice lines were good multi-environment germinators, and this percentage was lower in the dry season (3%, 42 lines). The good multi-environment germinators were *indicas* (33 lines) and *japonicas* (32 lines) in the wet season and *indicas* (31 lines) in the dry season (Supplemental Figure S1D and Supplemental Table S1). Because the best multi-environment germinators were rice lines from *indica* subpopulations, we focused analysis on the 1,094 (wet season) and 850 (dry season) *indica* lines.

### GWAS showed that relative germination is a polygenic trait

**Figure 1:**
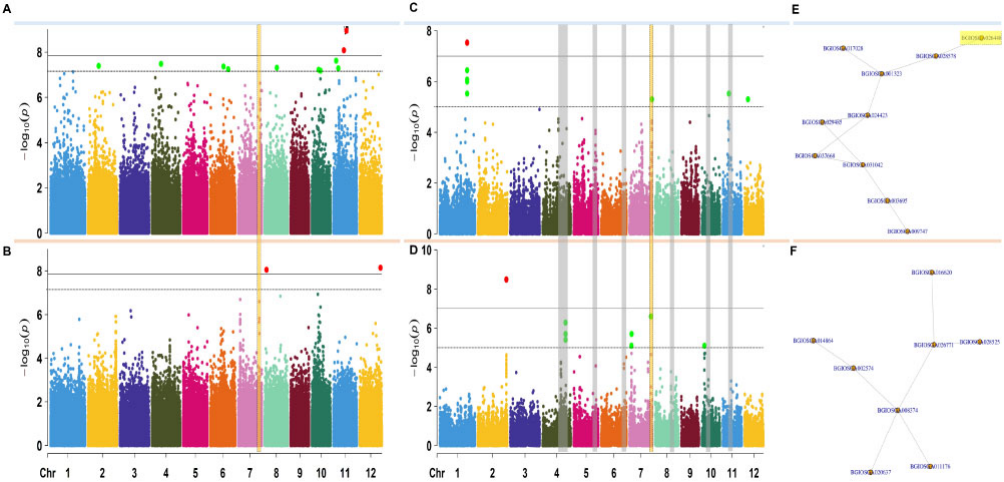
Genome wide association and post-GWAS results performed on the relative germination from *indica* subpopulation varieties from the 3,000 genomes panel. A, Manhattan plots of GWAS results for wet and (B) dry seasons. C, Manhattan plots of MAGMA analyses performed on the relative germination and the SNP-to-Gene genomic data (35,280 genes) for the wet season, and (D) the dry season. E, Subnetworks with the highest score from the top 100 modules created by using dense module network search (dmGWAS) in R for wet season and (F) dry season. In GWAS Manhattan plots, each dot is a single nucleotide polymorphism, while in MAGMA Manhattan plots each dot is a gene. The horizontal lines in a and b are the thresholds for significant –log_10_(*P*-value/Number of markers). Bold line: −log_10_(0.01/Number of markers) and dashed line: −log_10_(0.05/Number of markers). The horizontal lines in c and d are the thresholds for significant –log_10_(*P*-value*). Bold line: −log_10_(1e-7) and dashed line: −log_10_(1e-5). SNPs within a chromosome are colored as follows: chromosome 1-blue, 2-yellow, 3-purple, 4-darkgreen, 5-fuscia, 6-orange, 7-pink, 8-bluegreen, 9-brown, 10-lightgreen, 11-blue, and 12-yellow. In yellow box, chromosome region of interest and gene of interest within region. *P-values were corrected for multiple testing using permutation in MAGMA.

We integrated relative germination data by performing two *indica-*focused GWAS for wet (square root transformed) and dry seasons, using 693,502 SNPs generated from the IRRI 6.5 million SNP database (Mansueto et al., 2016). In addition to the kinship matrix in wet and dry seasons, we use age of seeds (Supplemental Table S2) as a covariate in our model for the wet season (seeds from the dry season were all the same age). GWAS results showed 475 SNPs (*P* <10^−5^; Figure 1) or 210 SNPs (FDR-adjusted *P* <0.05) and 176 SNPs (*P* <10^−5^; Figure 1) or 7 SNPs (FDR-adjusted *P* <0.05) with highly significant association values in the wet and dry season, respectively. Our GWAS results showed several associated SNPs with modest phenotypic effects in the wet and dry season (Supplemental Table S2). However, there were six potential QTL regions across seasons with hundreds of genes potentially associated to the trait on chromosomes 4, 5, 6, 7, 8, 10, and 11 (Figure 1—colored gray and yellow). Due to the high number of associated SNPs with small effects from multiple chromosomal regions (Boyle et al., 2017; Liu et al., 2018), we concluded that relative AG is a polygenic trait, with little seasonal overlap of chromosomal regions with significantly associated SNPs.

### Post-GWAS pipeline identified genes significantly associated with relative germination

GWAS analysis identified several potential candidate genes. To select genes and pathways for further study, we performed a generalized gene analysis of SNPs from both seasons using MAGMA (de Leeuw et al., 2015). These post-GWAS results showed sharper signals of significantly associated genes in the wet season, with 36 genes in the wet season compared to 27 genes in the dry season with genome-wide significance level ≤0.0001 (Figure 1). This analysis showed one region on chromosome 7 with an effect across seasons. MAGMA results were then merged with a protein–protein interaction (PPI) network (MH63, *indica*; Song et al., 2018), allowing integration of biological PPIs as links in the network. Within the two season-linked networks (Supplemental Table S2), the top connected protein modules with the highest additive MAGMA scores (Figure 1) were BGIOSGA017028 for the wet season (*Z*_m_ = 9.41; Figure 1) and BGIOSGA028525 (*Z*_m_ = 8.61; Figure 1) for the dry season. We looked at the chromosomal location of each node in the two season-linked subnetworks and narrowed this signal down to one protein: BGIOSGA026448/Os07g0693800 (Chr7: 27,641,164–27,653,064) on chromosome 7. The genomic location of this protein overlaps the region that has several significant genes in the MAGMA-GWAS results for both seasons (Figure 1). We concluded that there is a high association between the relative germination trait and this region on chromosome 7.

To link the gene analyses results from MAGMA of the wet and dry season, we performed a meta-analysis that takes into consideration linkage disequilibrium (LD) by linking the SNPs in 10-kb windows to the corresponding genes in those regions from the *indica* reference genome (ASM465v1; de Leeuw et al., 2015). The analysis showed 483 genes significantly associated with a *P* <0.01 (Supplemental Figure S2). These 483 genes were selected for further gene ontology (GO) characterization using AgriGOV2.0 (Du et al., 2010; Tian et al., 2017). Using the *O. sativa indica* (Rice TIGR gene model; Kawahara et al., 2013) annotation, we determined that of the 483 genes only 300 had GO annotations. Out of these, we found 116 GO terms that were significantly enriched (Supplemental Figure S2). Among these significant GO terms were: fatty acid and carbon metabolism, response to abiotic stimulus, negative regulation of gene expression, and methylation (Supplemental Figure S2 and Supplemental Table S3). The enrichment results showed that the genes highly correlated to germination under flooding are within the *response to stimuli* GO term and take part in gene regulation and methylation pathways. Among the genes in the methylation GO category, four genes are on chromosome 7 (BGIOSGA026447/Os07g0693700; BGIOSGA026441/Os07g0692500; BGIOSGA024323/Os07g0475800; BGIOSGA024320/Os07g0476200). Of these proteins, we focused on *CLASSY 1* (*CLSY1*, BGIOSGA026441/LOC_Os07g49210/Os07g0692600, Chromosome 7: 29,473,405-29,475,312; UniProt ID: Q0D3D6) to test for functional association with the AG trait using FN-mutants (Fast neutron mutants) from the UC Davis mutant library (Li et al., 2017). We focused our functional analyses on *CLSY1* because it fulfilled the following set of criteria: significant association to the trait in GWAS and/or post-GWAS; was part of the gene regulatory ontology term; and membership in the dmGWAS top subnetwork.

In addition to mutants and SNP-by-SNP GWAS, we used a different association genetics approach to show that the *CLSY1* genomic region predicts AG. Here, we used six genome-wide SNP principal component axes to control for genomic background (e.g. Price et al. (2006)). To represent possible effects of the target region, we used the *indica* genome model BGIOSGA026441 (Chr7: 27,578,735–27,588,520 nt), extracting three principal components that summarize local SNP variation in this region. Using multiple regression, we tested the relation between these nine principal components and the phenotype data from wet and dry seasons (Supplemental Table S2). Results showed that the phenotype in the wet season was significantly predicted by PC2 from the *CLSY1* genomic region on Chromosome 7 (*P *<* *6.806e-07; Table 1). This third approach is not fully independent of SNP-by-SNP GWAS, but finding a positive signal in both analyses reinforces our focus on this target region.

**Table 1:** ANOVA results for the relative germination in the wet and dry seasons with the three principal component loadings for the SNP composition from the region of interest (PC_Region) and six principal component loadings for the whole SNP genome data for *indica* (PCA_Genome).

*Wet season*						
**Term**	**df**	**sumsq**	**Meansq**	**Statistic**	***P*-value**	**P-value adj**

PC_Region1	1	3.33E-02	0.0332838	0.440204	5.07E-01	1.00E + 00
PC_Region2	1	2.25E + 00	2.2468779	29.716702	6.19E-08	**6.81E-07**
PC_Region3	1	1.14E-01	0.1140305	1.508142	2.20E-01	1.00E + 00
PCA_Genome1	1	9.13E-02	0.0913286	1.207892	2.72E-01	1.00E + 00
PCA_Genome2	1	5.69E-03	0.0056875	0.075221	7.84E-01	1.00E + 00
PCA_Genome3	1	3.05E-01	0.3049699	4.033463	4.49E-02	4.93E-01
PCA_Genome4	1	2.84E-01	0.2843742	3.761069	5.27E-02	5.80E-01
PCA_Genome5	1	1.91E-04	0.0001913	0.00253	9.60E-01	1.00E + 00
PCA_Genome6	1	3.53E-03	0.0035253	0.046625	8.29E-01	1.00E + 00
age	1	1.05E-04	0.0001046	0.001383	9.70E-01	1.00E + 00
Residuals	1086	8.21E + 01	0.0756099			

*Dry season*						
**Term**	**df**	**sumsq**	**Meansq**	**Statistic**	**P-value**	**P-value adj**

PC_Region1	1	0.051327	0.051327	2.259	1.33E-01	1.00E + 00
PC_Region2	1	0.086395	0.086395	3.802	5.15E-02	5.15E-01
PC_Regopm3	1	0.002954	0.002954	0.13	7.19E-01	1.00E + 00
PCA_Genome1	1	0.062416	0.062416	2.747	9.78E-02	9.78E-01
PCA_Genome2	1	0.177507	0.177507	7.811	5.31E-03	5.31E-02
PCA_Genome3	1	0.439879	0.439879	19.356	1.22E-05	1.22E-04
PCA_Genome4	1	0.007953	0.007953	0.35	5.54E-01	1.00E + 00
PCA_Genome5	1	0.076761	0.076761	3.378	6.64E-02	6.64E-01
PCA_Genome6	1	0.150507	0.150507	6.623	1.02E-02	1.02E-01
Residuals	840	19.089394	0.022725			

P-value adjusted by using Bonferroni correction. Bold value represents statistically significant p-value adjusted < 0.05.

**Figure 2:**
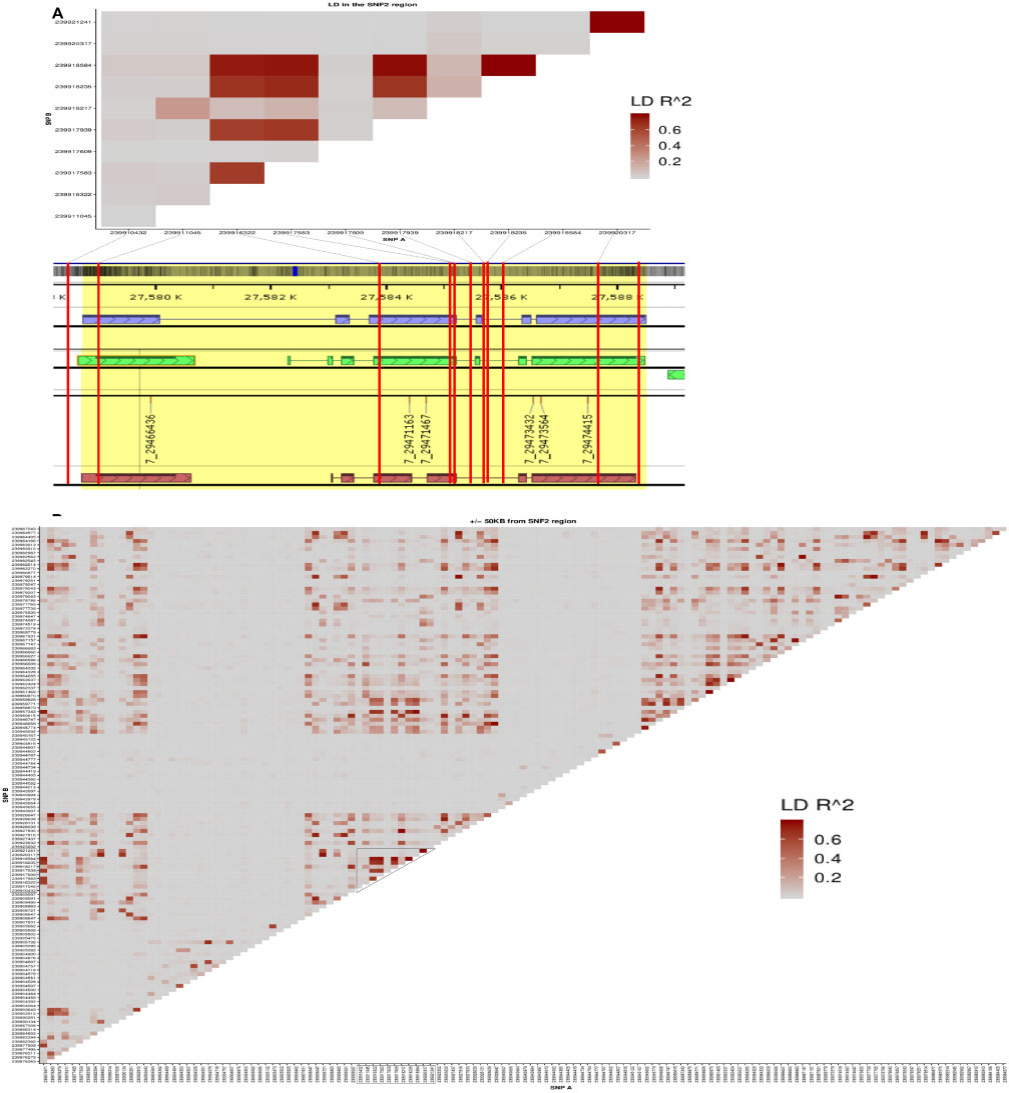
LD plot of (A) the 11 SNPs in the region of interest and gene models from the Oryza *indica* reference ASM465V1. Blue rectangles indicate BGI gene model for BGIOSGA026441. Oryza *japonica* reference MSU_osa1r7. Green rectangles indicate gene model for Gnomon: XM_015789529.1/LOC9266851 and XM_015790931.1/LOC4344376. Oryza *japonica* reference MSU_osa1r7. Brown rectangles indicate gene model for FGENESH: gene7_4409 and gene7_4410. LD values were calculated for the SNPs within the region of interest. B, The 50-kb up- and downstream genomic region around the target region. Gene models were taken from Persephone web software. Color in LD plot indicates LD *r^2^* (Red: 1 = *r^2^*, gray: 0 = *r^2^*).

We also examined LD in the region of interest annotated for *indica* rice background near BGIOSGA026441 (Chromosome 7: 27,578,735–27,588,520 annotation: ASM465V1; *japonica* background LOC_Os07g49210 annotation: MSU; Figure 2). Results show patchy, variable levels of LD in the *CLSY1* locus and flanking regions. SNPs within the methyltransferase gene (BGIOSGA026441/Os07g0692401) show little LD with the SNPs in the *CLSY1* locus (BGIOSGA026441/Os07g0692600). Several SNPs within *CLSY1* tend to be in high LD with each other. Furthermore, we checked whether the down- and upstream genomic regions were in LD with the 11 SNPs of interest. LD analysis of the ±50-kb regions showed that the *CLSY1* and *methyltransferase* regions are in LD with SNPs up- and downstream from them (Figure 2; Supplemental Figure S2). Two regions down- and upstream from the *methyltransferase* and *CLSY1* genes are in low LD, respectively (Figure 2). We conclude that our gene of interest shows moderate LD with nearby SNPs in the region and with SNPs up- and downstream. Thus, our focus on *CLSY1* is reinforced by its low LD with the methyltransferase gene, in combination with GWAS and post-GWAS analyses.

### Candidate mutants are taller than WT plants under flooding

Considering the genes of interest determined by using GWAS and post-GWAS, we selected mutant lines for functional genomics studies from the UC Davis collection, (Li et al., 2017). To identify the functional association between the genes of interest and germination under flooding environment, we performed a preliminary screening, focusing on mutants FN-559-S (https://kitbase.ucdavis.edu/search_result?mutant_id=FN559-S) and FN-544-S (https://kitbase.ucdavis.edu/search_result?mutant_id=FN544-S). Both mutants carry a mutation in the *CLSY1* region, Chromosome 7: 27,578,735–27,588,520 (*indica* background BGIOSGA026441, annotation: ASM465V1; *japonica* background LOC_Os07g49210 annotation: MSU). FN-559-S carries an 11-bp deletion in chromosome 7: 29,473,577–29,473,588. FN-544-S carries a 7,596-bp inversion in chromosome 7: 29,473,094–29,480,690. Within this region, in *japonica* background (Nipponbare annotated genome), there are three annotated genes: Os07g0692401, Os07g0692500, and Os07g0692600 (Chromosome 7: 29,465,173–29,475,499; annotation: IRGSP-v1.9).

**Figure 3:**
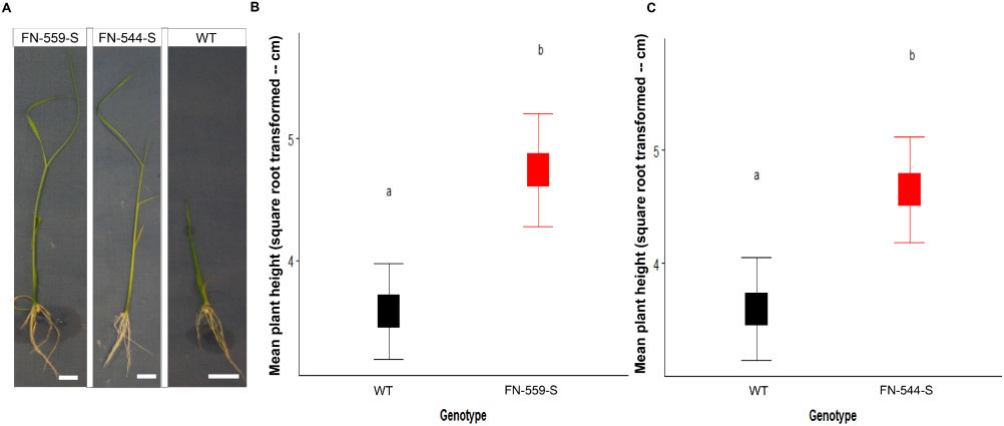
Phenotype of the mutant lines and WT under flooding conditions. A, Pictures of FN-559-S, FN-544-S, and WT plants after flooding treatments. White bar = 19.05 mm. B, Least-square means of square root transformed plant height at Day 14 in the flooding environment. FN-559-S (red) and WT (black). C, Square root of the least-square means of FN-544-S (red) and WT (black) at Day 14 in flooding environment. Mutants FN-559-S and FN-544-S are compared to WT by one-way ANOVA. Letters above square plots indicate the honestly significant difference–Tukey’s test results with *P* < 0.05. Error bars represent standard errors.

Following GWAS, we performed two analyses to determine if the gene affected in FN-559-S or FN-544-S influences AG. The first line of evidence was a phenotypic analysis using FN-559-S mutant seeds from the third generation by single seed descent). Phenotypic results of FN-559-S showed a 50% increase in plant height under flooding compared to wild-type (WT; LSmeans of height were 22.49 cm for heterozygous FN-559-S genotype and 12.82 cm for WT–Kitaake; *N*_mutant_ = 14, *N*_WT_ = 28, *P* = 0.01; Figure 3). The frequency of *clsy1* (M1-559) homozygous mutants was too low to have at least three biological replicates per treatment. Thus, homozygous mutants were excluded from statistical analyses. We did not find significant differences in the day of germination between FN-559-S and WT–Kitaake (Supplemental Figure S3), meaning that mutant and WT genotypes germinated at the same time. Also, *clsy1* (M1-559) showed a longer root phenotype (>50%) than WT–Kitaake (*N*_mutant_ = 14, *N*_WT_ = 28, *P* = 0.005; Supplemental Figure S3).

The second line of evidence was a phenotypic analysis performed using mutant *clsy1* (M2-544 mutant seeds from the third generation by single-seed descent) in flooding environments. Phenotypic results for FN-544-S showed a 50% increase in plant height compared to WT under flooding (LSmeans of height were 21.62 cm for heterozygous FN-544-S and 12.92 cm for WT–Kitaake; *N*_mutant_ = 25, *N*_WT_ = 28, *P* = 0.02; Figure 3). We were unable to test the homozygous FN-544-S because none of the tested progeny had this genotype. We hypothesize that this was due to embryonic lethality in the homozygous FN-544-S. We did not find differences in the day of germination between FN-544-S and WT–Kitaake (Supplemental Figure S3). Also, FN-544-S had a longer root phenotype (>50%) than WT–Kitaake (*N*_mutant_ = 25, *N*_WT_ = 28, *P* = 0.007; Supplemental Figure S3). Thus, these two independent mutants showed that FN-559-S and FN-544-S mutants are almost double in height compared to WT–Kitaake in a flooding environment, meaning that when one allele of this gene is mutated it positively influences height during flooding, facilitating seedling establishment under a stressful environment.

### FN-559-S reduces gene expression of a linked methyltransferase in the control environment

**Figure 4:**
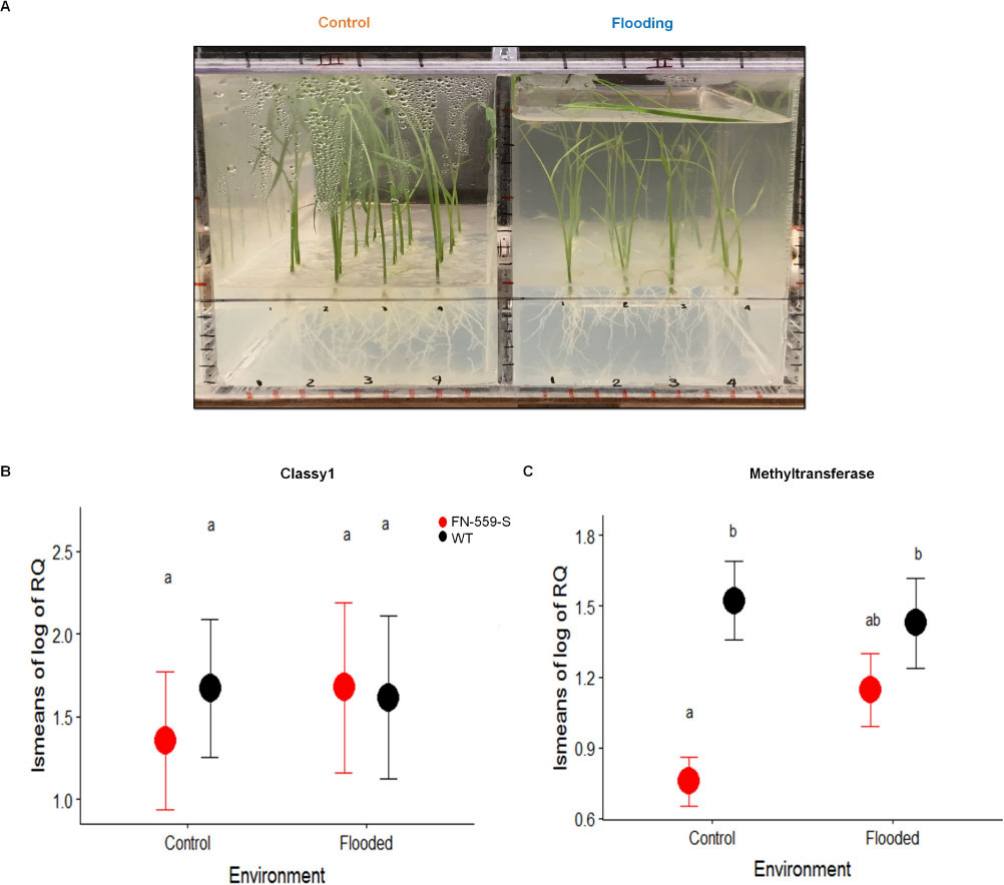
Phenotyping set-up and gene expression of FN-559-S and WT under control and flooding conditions. A, Pictures of experimental set-up used for plants under control and flooding treatments at Day 14 after planting. B, Log_10_ relative gene expression of *CLASSY1* gene in FN-559-S (red) and WT (black) at Day 14 in control and flooding environment. C, Log_10_ relative gene expression of methyl transferase gene in FN-559-S (red) and WT (black) at Day 14 after planting in control and flooding environment. In (B) and (C), Tukey–honestly significant difference test results with *P* < 0.05 are shown by letters above means. Bars represent standard errors.

To determine the molecular mechanisms for enhanced plant height in flooding environments in FN-559-S, we performed gene expression analysis of *CLSY1* (XM_015789529.1/LOC9266851) and the methyltransferase gene present in the chromosomal region of interest. Relative *CLSY1* gene expression showed no differences between heterozygous FN-559-S mutant and WT-Kitaake under flooding or control conditions (*P*_Treatment_ = 0.81, *P*_genotype_ = 0.74; Figure 4). This result was expected because the deletion in the FN-559-S mutant allowed the synthesis of mRNA that contained a premature stop codon that could lead to a ∼90% change of the original protein sequence (Supplemental material 2). The *CLSY1* gene is part of the RdDm epigenetic pathway in rice (Hu et al., 2013) and is an ortholog of the *CLASSY1* gene in Arabidopsis (Zhou et al., 2018). A possible effect of FN-559-S downstream of CLSY1 could involve changes in methylation and gene expression profiles. In the control environment, relative expression of the methyltransferase gene was significantly lower (*N*_mutant_ = 4, *N*_WT_ = 6, *P*_genotype_ = 0.002) in FN-559-S compared to Kitaake (Figure 4). Under nonflooding conditions, gene expression of the methyltransferase was lower in the mutant compared to WT–Kitaake, and the FN-559-S deletion mutation falls within the *CLSY1* gene (Li et al., 2017). Although we did not find statistically significant differences in expression of this methyltransferase gene under flooded conditions, we hypothesize that truncation in the *CLSY1* gene may lead to changes of whole-genome methylation profiles under control environments. We propose that truncation of the *CLSY1* gene in rice may alter the RNA-guided DNA methylation pathway that leads to enhanced plant height in flooded environments due to changes in methylation profiles and overall changes in gene expression. The molecular mechanisms behind this interaction are not elucidated here and will need further analysis.

### Whole-genome methylation profiles differ between FN-559-S and WT–Kitaake under flooding

**Figure 5:**
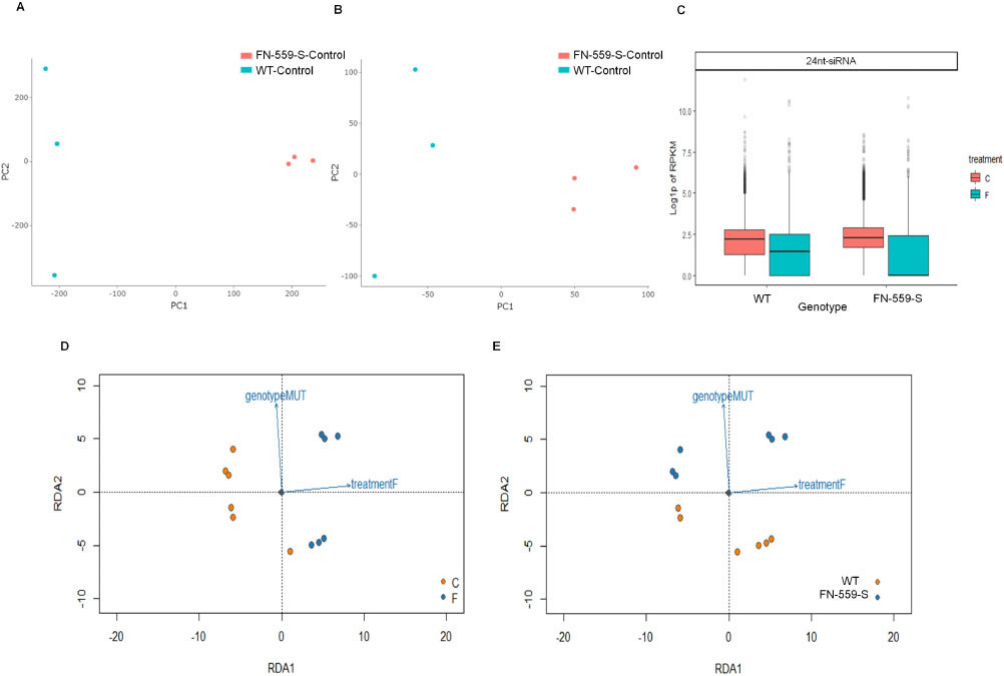
CpG Methylation profiles from leaves using principal component analysis of the CpG sites from FN-559-S and WT–kitaake under (A) control and (B) flooded environments. Color points represent genotype, FN-559-S: salmon and WT: blue. Profiles of 24nt-siRNA leaves from WT and FN-559-S under control and flooded treatments. C, Log_10_(*P*-value) of RPKM, normalized by using DEseq dispersion coefficients and size factor normalization. FN-559-S siRNA profiles are different from WT. Figure faceted by dicercall size generated using Shortstack, error bars show the standard error, blue color indicates flooded treatment and salmon color indicates control treatment. Diamond shapes are FN-559-S and circles are WT. D, Profile of RDA model using 24nt-siRNA RPKM data from leaf tissue. Color of dots represents treatment variable (Control: salmon; flooded: blue), (E) color of dots represents treatment variable (WT: salmon; FN-559-S: blue) Rotated axes are the scores of RDA1 and RDA2 model fit.

To test if the whole-genome methylation profiles were affected by the deletion of the *CLSY1* gene, we performed genome-wide bisulfate sequencing on FN-559-S and WT–Kitaake under control and flooded environments. In all samples and treatments, over 40% of methylation events were CpG, whereas ∼20% and ∼3% were CHG and CHH types (Supplemental Table S4). There were CpG methylation profile differences between WT and heterozygous FN-559-S in the control and flooded environments (Figure 5). The majority of CpG differentially methylated sites (*q*-values <0.05 and differential methylation level ≥25%; Wang, 2010) overlapped promoter and intergenic regions of loci (Supplemental Table S5). We compared the percentage CpG methylation of FN-559-S and WT in the flooded environment and detected 758 differentially methylated loci (DML) events. Because there can be multiple CpG methylation events within the same locus, the 758 events were within 395 unique loci (Supplemental Table S6). The quality of the methylation data used was above 30 QC-score and the average unique mapping rate was 73.57% (Supplemental Table S6), which is within the range of recently published bisulfate sequencing data (Zhou et al., 2018; Wu et al., 2020).

We performed a metabolic pathway analysis (gramene.org) on the 395 DML. This analysis linked 10 of these loci to well-characterized metabolic pathways and found 21 over-represented pathways. Among these pathways were regulation of seed size and seed development (Supplemental Table S7). Among the genes that belong to both of these metabolic pathways, two genes had multiple methylation events: Os02g0244100 (*E3 ubiquitin-protein ligase DA2*, *q*-value_Event-1_ = 0.003, methylation difference_Event-1_ = 33.3; *q*-value _Event-2_ = 6.13 × 10^−06^, methylation difference _Event-2_ = 61.25; Supplemental Table S6 and Supplemental Figure S4) and Os11g0523800 (Auxin response factor 2; *q*-value _Event-1_ = 7.72 × 10^−08^, methylation difference _Event-1_ = 71.43; *q*-value _Event-2_ = 6.43 × 10^−08^, methylation difference _Event-2_ = 75.86; Supplemental Table S6 and Supplemental Figure S4). In this analysis, the mutant was the “control group”, the WT was the “treatment group”, and the methylation differences were determined as the treatment minus control difference. Both genes showed significant differential methylation with higher methylation percentages in WT compared to FN-559-S flooded (high positive value). A positive differential methylation value indicates hypomethylation in the mutant.

The *Auxin response factor 2* (Os11g0523800) gene showed significantly higher transcript levels in *clsy1* (M1-559) control compared to FN-559-S flooded, showed no gene expression differences compared to WT flooded (Supplemental Figure S4), and had higher methylation levels in WT flooded compared to FN-559-S flooded (Supplemental Table S6). Although we hypothesized that a deletion in *CLSY1* could lead to reduced methylation and higher gene expression due to de-regulation of the RdDm pathway, those hypomethylated regions might not all be gene-coding areas (Zhou et al., 2018). Perhaps, repression of seed development is deactivated under stress environments by other epigenetic pathways besides RdDm that do not involve *CLSY1* and methylation events (Kumar et al., 2018). Also, there may be other regulatory pathways that are controlling the expression of genes involved in seed development (Iwasaki et al., 2019), especially under stressful environments. Thus, gene expression is not suppressed on these stress-related response genes and pathways in our *CLSY1* mutant.

### Small RNA-seq and RNA-seq profiles differ between FN-559-S and WT–Kitaake in flooded and control environments

In Arabidopsis, *CLSY1* influences locus-specific control of 24-nt small-interfering RNA (siRNA) production (Zhou et al., 2018). To test if the deletion in *CLSY1* changed siRNA production and overall gene expression in leaves under flooded conditions, we performed small RNA-seq and RNA-seq analysis on FN-559-S and WT–Kitaake under control and flooded environments. The small RNA-seq data detected 32,787 genomic clusters, with 79% of these clusters classified as 24-nt siRNAs (24-nt siRNAs: 25,878; 23-nt RNAs: 507; 22-nt RNAs: 583; 21-nt RNAs: 1094; 20-nt RNAs: 255; Supplemental Figure S5). The reads per kilobase of transcript per million mapped (RPKM) from the 24-nt siRNA clusters showed a significant linear relationship with the treatment variable under a multivariate redundancy analysis (RDA) model (Legendre and Legendre, 1998; *P* = 0.02; Figure 5, D and E), which predicted ∼14% of RPKM data variation (Figure 5; Supplemental Material 1). We determined from the angle between the response variable vectors in the RDA figures that *clsy1* (M1-559) and flooded treatment showed no significant covariation (Cos 90° = 0, Figure 5). The analysis of variance (ANOVA) of the multivariate model indicates that the 24-nt siRNA clusters covary only with treatment (*P* = 0.009, 999 permutations performed for *P*-value adjustment), meaning that there will be significant changes in the 24-nt siRNA profiles in flooded conditions. We hypothesize that 24-nt siRNA RPKM profiles did not covary with the mutant variable because production of 24-nt siRNAs may be cluster-specific under flooded environments, and the overall 24-nt RNA RPKM profiles may not reflect the specificity in our *CLSY1* mutant. Also, the heterozygous deletion genotype in *CLSY1* is not dominant, and alternative epigenetic pathways (Kumar et al., 2018; Iwasaki et al., 2019) might be involved in the production of 24-nt RNAs.

**Figure 6:**
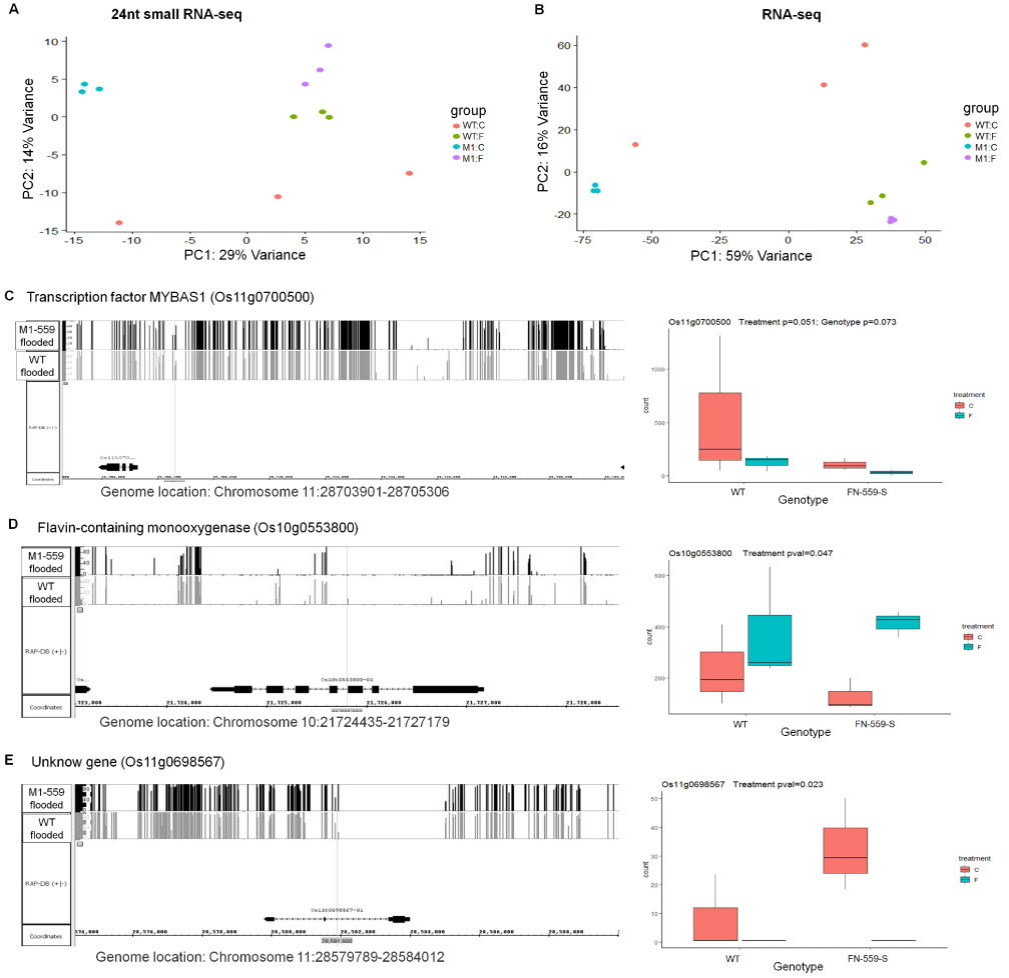
Profiles of 24nt-siRNA and RNA levels in leaves from WT and FN-559-S under control and flooded treatments. A, Principal components 24nt-siRNA-seq of rlog transformed counts from normalized counts. B, Principal component analysis of RNA-seq of rlog transformed counts from normalized counts. Point colors are salmon: WT–control, green: WT-flooded, blue: FN-559-S*-*control and purple: FN-559-S-flooded. Normalized counts were obtained after calculating dispersion coefficients and size factors on each library using DESeq. Profiles of DML methylation, a nearly significantly differential expressed 24nt-siRNA cluster and significantly differentially expressed genes. C, Os11g0700500, (D) Os10g0553800, (E) Os11g0698567. In each panel: on the right are methylation profiles of DML from leaves of FN-559-S (black bars) and WT (gray bars) under flooded environment. On the left, gene expression profiles from leaves of FN-559-S and WT under flooded and control environment. Genome annotation is RAP-DB 2011 version. Counts from rlog transformed count data from additive model. Error bars show the standard error. Blue indicates flooded treatment and salmon indicates control treatment. Normalized counts were obtained after calculating dispersion coefficients and size factors for each library using DESeq. Wald-*P*-value was calculated using DESeq additive model of RNA count levels.

To test if cluster-specific expression of 24-nt siRNA was affected by flooded conditions, we performed gene expression analysis on the small-RNA-seq cluster counts. Our small-RNA-seq results showed that there were differences in cluster-specific expression between FN-559-S and WT under control and flooded conditions. Of the 25,878 24-nt siRNA clusters, 27 showed significant differences in expression in contrasting treatments (adjusted *P* <0.05; Supplemental Table S8) and 13 when contrasting genotypes (adjusted *P* ≤0.05; Supplemental Table S8). To determine if FN-559-S changed gene expression profiles, we performed RNA-seq analysis. RNA-seq results showed that there were significant differences in gene expression between FN-559-S and WT under control and flooded conditions (Figure 6). We detected expression of 29,644 genes, among which 3,688 were differentially expressed in contrasting treatments, with 1,868 upregulated and 1,820 downregulated (adjusted *P* <0.05; Supplemental Table S9). Also, 608 genes were differentially expressed between genotypes: 241 were upregulated and 367 were downregulated (adjusted *P* ≤0.05; Supplemental Table S9).

We looked at the genomic region of interest on chromosome 7 (27,578,735–27,588,520) and found no DML and no small RNA-seq clusters within that region. Also, there were no significant differences in gene expression of *CLSY1*, whereas the methyltransferase gene expression profile showed decreased counts in FN-559-S under control and flooded conditions (Supplemental Figure S6). Controlling for multiple tests, we found three genes that had differential methylation, a nearby differentially expressed 24-nt siRNA cluster, and differential gene expression of Os11g0700500 (*Transcription factor MYBAS1*; *q*-value <0.05 and methylation difference >40%; Figure 6; Supplemental Tables S6 and S9), Os10g0553800 (*Flavin-containing monooxygenase*; *q*-value <0.05 and methylation difference >35%; Figure 6; Supplemental Tables S6 and S9), and Os11g0698567 (Unknown gene; *q*-value <0.05 and methylation difference >45%; Figure 6; Supplemental Tables S6 and S9). Their role in the downstream metabolic and physiological responses to flooding have not been characterized. Nevertheless, due to their methylation profile, siRNA-link, and gene expression differences, these genes might play an important role in rice germination and seedling establishment during floods.

## Discussion

We found that *indica* rice genotypes tend to have a stable phenotypic response across water regimes. Hence, natural variation within *indica* could hold the key to understanding the genetics leading to a stable AG trait. In wet versus dry seasons, there are marked environmental differences such as lower solar radiation, higher precipitation averages, and lower temperatures during the wet season (Yoshida, 1977; Yang et al., 2008; Silva et al., 2017), which could lead to different plant responses to flooding. We hypothesized that several of the *indica* rice landraces tend to be grown in tropical and subtropical climates with wet season (d’Alpoim Guedes et al., 2015), which could influence their status as good multi-environment germinators. Further studies will be necessary to test this hypothesis. GWAS and gene set analyses showed six potential QTL regions on chromosomes 4, 5, 6, 7, 8, 10, and 11 (Figure 1—colored gray and yellow). These regions contain several genes that show association with the trait across seasons. Based on the high number of associated SNPs with small effects from multiple chromosomal regions (Boyle et al., 2017; Liu et al., 2018), we conclude that relative AG is a polygenic trait. These results were expected because there are several physiological responses in domesticated rice (Voesenek and Bailey-Serres, 2013, 2015) that have been linked to seedling survival under anaerobic environments (He and Yang, 2013; Miro and Ismail, 2013; Toledo et al., 2015). These responses include the above-mentioned LOES and LOQS (Voesenek and Bailey-Serres, 2015). Thus, the genetic architecture of germination under flooding appears polygenic in nature.

As often happens, our GWAS analysis identified several potential candidate genes that were worthy of further study. At that point, our post-GWAS approach leveraged techniques from human genetics (de Leeuw et al., 2015) and network biology (Wang et al., 2015) to identify specific rice mutants with functional effects on AG. These include genes involved in fatty acid metabolism, ethylene perception, sugar metabolism, regulation of gene expression, and epigenetics. We identified a gene regulatory pathway that influences the AG trait through RdDm processes and determined that the *CLSY1* gene contributes to the capacity of rice to germinate under anaerobic conditions. In Arabidopsis, *CLSY1* is a key gene in the RdDm pathway, influencing locus-specific control of 24-nt siRNA production, methylation, and gene expression profiles (Zhou et al., 2018). Under AG conditions, our heterozygous FN-559-S mutant showed significantly greater plant height than WT. In addition, under nonflooding environments the *CLSY1* rice mutant showed lower gene expression of a methyltransferase compared to WT (Figure 4). The interaction between *CLSY1* and the methyltransferase might be linked to a PPI disruption in heterozygous *CLSY1* mutants, and further research to elucidate this interaction would be useful. Despite not finding differences in the tested methyltransferase gene expression under flooded conditions, we detected differences in methylation at the whole-genome level (Figure 5), showing that that flooding alters the methylation profiles in the *CLSY1* mutant, as well as reducing RPKM profiles of 24-nt siRNAs (Figure 5). All these changes point to a key role of RdDm/*CLSY1* in AG responses that causes physiological changes advantageous for seed germination and seedling establishment under anoxic environments. It is possible that *CLSY1* differentially methylated regions might not all be within gene coding areas (Zhou et al., 2018), and the molecular mechanisms and impact on the phenotype will need further investigation.

In the FN-559-S mutant, we found genes that had differential methylation profiles, were close to a region with a differentially expressed siRNA cluster, and showed differential expression profiles. These genes were involved with auxin processes and plant growth, meaning that our mutant could alter multiple down-stream pathways that have important effects on rice physiological responses to flooding. Downstream changes caused by differential methylation and siRNA variation can lead to gene expression differences in the mutant under flooded compared to control environments. Several of these genes seem to be involved in the Calvin cycle, TCA pathways, and other metabolic processes. The role of the TCA pathway under AG conditions in seedlings has been studied at length; under anaerobic conditions the plant switches from the TCA cycle to fermentative metabolism to produce ATP (Magneschi and Perata, 2009; Miro and Ismail, 2013; Ray et al., 2016). The production of ATP under anoxic conditions comes from a multi-step process in which sucrose is cleaved to generate pyruvate for the oxygen-dependent TCA cycle (Ray et al., 2016). We found several SNPs and genes that might be part of these metabolic processes. However, the molecular mechanisms are not fully understood (Takahashi et al., 2014) and are not the primary aim of this research.

Our heterozygous FN-559-S showed significant differences in its small RNA, whole-genome methylation, and gene expression profiles compared to WT. In this mutant, the overall gene expression profiles suffered higher deregulation than the small-RNA cluster-specific expression. Taking together, our physiological and omics results and the low number of differentially expressed clusters in small-RNA-seq data compared to RNA-seq data, we propose that there is a tight regulation of changes involving the RdDm processes, indicating that small changes in this pathway can lead to a marked effect on gene expression and physiological responses in the plant under both control and flooded environments.

## Conclusions

We conclude that AG is a complex polygenic trait. Our post-GWAS approach leveraged techniques from human genetics (de Leeuw et al., 2015) and network biology (Wang et al., 2015) to identify specific rice mutants with effects on AG. Several approaches support functional effects of *CLSY1* on AG. We propose that a mutation in the *CLSY1* gene in rice influences the RdDm pathway leading to changes in methylation profiles and gene expression patterns, causing enhanced survival of seeds under anoxia and greater seedling establishment in flooded environments. Our work furthers the knowledge and understanding of pathways influencing AG. Although the *CLSY1* alleles in the GWAS population have moderate effects, it is possible that epistasis might influence effects of this locus, resulting in larger allelic effects in some genetic backgrounds. We suggest that these post-GWAS approaches can help prioritize polygenic candidates for other traits and agricultural challenges.

## Methods

*Experimental design and screenhouse phenotyping.* Counts of germinated plants were recorded for the 2017 wet and 2018 dry season. 2,700 rice genotypes (20 seeds per genotype, totaling 109,440 seeds) were screened in the wet season whereas 1,509 rice genotypes (30 seeds per genotype, 91,800 total seeds) were screened in the dry season. There are marked differences between seasons, such as lower solar radiation, higher precipitation averages, and lower temperatures during the wet season (Yoshida, 1977; Yang et al., 2008; Silva et al., 2017). The first experiment was performed in the wet season with seeds available in the International Rice Genebank at the International Rice Research Institute. Subsequently, a seed increase was performed using seeds from the control environments in the wet season. The newly harvested seeds were used for the dry season experiment. In both seasons, we used an incomplete randomized complete block (IRCB) design (Ireland, 2010) for the control and 8-cm flooded environment. IRCB is a partially balanced incomplete block design that uses checks (Controls) in each block to account for experimental variability instead of replicates of each data cell (Patterson and Williams, 1976). The rice genotypes used for checks were Ma Zhan (Red), Khao Hlan On, IR 42, and IR 64.

*Estimation of adjusted means*. To determine the adjusted means for the number of plants germinated we used the augmented randomized block experimental design model using the PBTools software V1.4 (http://bbi.irri.org/products). We calculated the adjusted means for each genotype used in flooded (AG) and control environments. The minimum number of germinating plants was 0 and the maximum was 20 in the wet season and 30 in the dry season. Any estimated mean above or below those limits was transformed to the closest limit number. We used the adjusted means of plant counts to determine the percentage of germination of the genotypes in each environment (Eq. 1). To join the percentage of germination in control and flooded environments into one physiological trait we used relative germination (Eq. 2<). All downstream analyses were performed using relative germination values.

$$\mathrm{Percentage}\;\mathrm{of}\;\mathrm{germination}\;\mathrm{in}\;\mathrm{each}\;\mathrm{environment}=\left(\frac{\mathrm{no}.\;\mathrm{of}\;\mathrm{plants}\;\mathrm{in}\;\mathrm{environment}}{20\;\mathrm{Wet}\;\mathrm{or}\;30\;\mathrm{Dry}\;\mathrm{season}}\right)*100\;(1)$$

$$\mathrm{Relative}\;\mathrm{germination}=\left(\frac{\mathrm{Percentage}\;\mathrm{of}\;\mathrm{germination}\;\mathrm{in}\;\mathrm{flooded}}{\;\mathrm{Percentage}\;\mathrm{of}\;\mathrm{germination}\;\mathrm{in}\;\mathrm{control}}\right)\;(2)$$

*GWAS*. We integrated the relative germination data from wet (square root transformed) and dry seasons with a 693,502 SNP database using GAPIT (Lipka et al., 2012). To run GWAS, we used the GAPIT generated kinship matrix; model selection flag was set to TRUE and we use seed age as a covariate for the wet season. The GWAS results from GAPIT in Supplemental Table S2 were corrected using the false-discovery rate method (Lipka et al., 2012). The Manhattan plot thresholds were graphed using GAPIT Bonferroni cut-off values (GAPIT function code; Lipka et al., 2012). Genotype SNP data for the 6.5 million SNPs were downloaded from the IRRI website (https://snp-seek.irri.org/; Mansueto et al., 2016); this SNP database was created by IRRI from the biallelic 3kRG 29mio SNP dataset by applying the following filtering criteria: missing calls per sample <31% (1 sample deleted, see .irem file), missing calls per variant <20%, and minor allele frequency per variant >1%. The SNP data were filtered by keeping only *indica* genotypes and SNPs with heterozygosity values ≤30%, then it was LD pruned by a 2-step procedure using PLINK v1.9 (Chang et al., 2015): (1) LD pruning with window size 10 kb, window step: 1 SNP, *R*^2^ threshold: 0.8, followed by (2) LD pruning with window size 500 SNPs, window step 1 SNP, *R*^2^ threshold 0.8. Two *indica-*focused GWAS were performed using 1,094 rice lines in the wet season and 850 rice lines in the dry season. Data was tested for normality prior to running GWAS; wet season data was transformed (Square root transformation) to fulfill normality.

*Post-GWAS MAGMA.* To identify candidate genes and pathways, a generalized gene analysis of the GWAS results from wet and dry seasons was performed using MAGMA (de Leeuw et al., 2015). LD was considered by linking the SNPs in 10-kb windows to the corresponding genes in those regions from the *indica* reference genome (ASM465v1) using gene models for protein-coding genes. The MAGMA-gene analysis used correlations among local SNP markers to aggregate SNPs with low to moderate effect for testing trait associations at the level of genes (de Leeuw et al., 2015). We performed two gene analysis with MAGMA using the SNP-wise model flag to calculate mean SNP association values per gene. This was done for the wet and the dry seasons, followed by genome-wide correction for multiple testing using permutation (de Leeuw et al., 2015). Then we performed MAGMA meta-analysis using the weighted Stouffer's *Z* method (Stouffer et al., 1949) to combine *P*-values from independent statistical tests. The meta-analysis results were used for GO and dmGWAS analysis.

*GO enrichment.* Singular GO enrichment analysis was performed on the genes from MAGMA meta-analysis with a *P*-adjusted value ≤0.01 by using the AgriGO V2.0 annotation tool (Du et al., 2010; Tian et al., 2017). The GO enrichment was computed using Fisher’s exact test and the pre-calculated background genes (Rice TIGR gene model) followed by the Yekutieli (FDR) multi-test adjustment method. Meta-analysis results showed 483 genes significantly associated (*P* <0.01) to relative germination. We used *O. sativa indica* as the species and the *TIGR* rice gene models (Kawahara et al., 2013) as the background (30,241 genes).*dmGWAS.* We linked all meta-analysis permuted *P*-values with a pre-built PPI network from the rice information gateway (MH63 *indica* line, http://rice.hzau.edu.cn/; Song et al., 2018) and performed a dense module search (dmGWAS R package; Wang et al., 2015) in R V3.9.0 (Team, 2015). To find dense network modules that have significantly associated genes, we created a network for the top 100 protein modules, selected the protein with the highest module dense score and created a subgraph that had the highest aggregated local maximum proportion of low *P*-values (Wang et al., 2015; Figure 6). The scored *z*-value used in dmGWAS was developed by ranking the highly connected subnetworks using a scoring system of protein *P*-values (Ideker et al., 2002). In our analysis, this subnetwork would be composed of modules with proteins whose genes have a low *P*-corrected value from the MAGMA meta-analysis, reflecting their association value to the AG trait. Network visualizations and annotations were added to each protein by importing the GO data from gramene.org using ensembl and biomart (accessed in 2018, https://plants.ensembl.org/Oryza_sativa/Info/Index, https://useast.ensembl.org/info/data/biomart/index.html).

*Phenotyping of mutant progeny*. To phenotype the mutants from single-seed descent, we used acrylic chambers (44 cm × 20 cm × 19 cm) with removable tops. For all phenotyping experiments, each chamber was a block and each block was divided into four compartments (11 cm × 20 cm × 19 cm); these compartments were the split-plots. In each compartment, we added 1 L of Yoshida’s solution (Yoshida et al., 1976) in 0.3% Gelzan (Caisson Labs, Smithfield, UT, USA) then randomized and placed 32 seeds (∼2 cm below surface of the media) of WT/Kitaake (*N* = 16), and *clsy1* (M1-559) or *clsy1* (M2-544) independent mutant genotypes (*N* = 16) in a grid of 4 × 9 cells (seeds spaced ∼2 cm from each other). Two of the compartments in each block were aerobic controls, and the other two were flooded with 3.5 L of distilled autoclaved water. The treatment positions in the chambers were swapped in each block. Mutants 554 and 559 were phenotyped in separate experiments, and each experiment was a randomized split-plot design with 108 Kitaake and 108 mutant seeds. Phenotyping traits were day of germination and plant height and root length by the last day of the experiment. ANOVA was performed using R V3.9.0 (Team, 2015) on plant height by implementing a linear mixed model using two fixed variables: genotype and treatment and two random variables as part of the split mode: block (chamber) and split factor (compartment).

*Analysis of variance and LD analyses.* To determine whether the gene regions of interest predict trait values, we performed ANCOVA on the phenotype data for the wet and dry season using the first three principal components of the 11 SNPs within the chromosomal region of interest (Chr7: 27,578,735–27,588,520; Supplemental Table S2). Seed age was included as a covariate in the wet season experiment. In the ANCOVA, the genomic background was controlled by using the first six principal components from the whole genotype data from *indica* lines used for the GWAS. Principal components for the region of interest and genome-wide SNPs were calculated using plink V1.90 (Chang et al., 2015). To determine correlations among the SNPs in the region of interest, we calculated the LD *r^2^* values for 11 SNPs (Chr7: 27,578,735–27,588,520) using plink V1.90 (Chang et al., 2015). Also, to determine correlations among the SNPs near the region of interest, we examined the 50-kb up- and downstream flanking regions (Chr7: 27,538,520–27,658,520). We calculated LD *r^2^* using plink V1.90 (Chang et al., 2015). All the LD values were graphed using R V3.9.0 and ggplot (Team, 2015).

*Genotyping of mutant progeny.* Leaf samples were taken from each plant used in the phenotyping experiments for FN-559-S and FN-544-S. DNA was extracted using QuickExtract™ Plant DNA Extraction Solution (Lucigen, Wisconsin, USA) following manufacturer instructions. To genotype mutant 554, we performed PCR targeting the parental gene (F: TGT TTT GTC CCG ACT TCT GA, R: GTC CAA GCT CCT CAT CCA GT) and mutated gene regions (F: GTC CAA GCT CCT CAT CCA GT, R: ACA GTA GAC TTT GCC TGC CT) following PCR conditions: Step 1: 95°C for 3 min, Step 2: 95°C for 15 s, 67°C (For mutant gene) or 57°C (For parental gene) for 30 s and 72°C for 45 s for 45 cycles, Step 3: 72°C for 10 min. The band patterns were visualized in a 3% agarose (Genesee Scientific) gel. To genotype mutant 559, we performed PCR using the following primer set (F: GTA AAA CGA CGG CCA GTA CAA GGA CAG ACC TGG ATG C, R: GCA GTG TTT TCC CAG AT, M13: [FAM]GTA AAA CGA CGG CCA GT, [HEX]GTA AAA CGA CGG CCA GT, [ROX]GTA AAA CGA CGG CCA GT) with the M13-dye tag system (Schuelke, 2000) and the following PCR conditions: Step 1: 95°C for 3 min, Step 2: 95°C for 15 s, 58°C for 30 s and 72°C for 45 s for 45 cycles, Step 3: 72°C for 10 min. We sent the M13-dye-labeled PCR product for fragment analysis (Eton, Durham, NC, USA) and detected size differences of mutant and WT amplified genomic region by using Free Peak Scanner^TM^ Software v1.0.

*RNA extraction, and reverse transcription quantitative PCR analysis.* Leaf samples (*N*_M1_ = 4, *N*_WT_ = 6) were ground using a ball-mill tissue grinder (Genogrinder 2000; SpexCentriprep Inc., Metuchen, NJ, USA) for 15 s at 2,000 strokes/min under liquid nitrogen. Sample RNA was extracted using Zymo^®^ following manufacturer instructions. RNA content was measured using a Nanodrop (ThermoFisher Scientific), and cDNA was made using High-Capacity cDNA Reverse Transcription Kit (ABI, Foster City, CA, USA) following manufacturer instructions. Reverse transcription quantitative PCR (RT-qPCR) analyses were done using primers for endogenous control Os11g26910 (F: ATC CTG GCC GCG AAC TA, R: CCA CTG GTT CTC CCT GC), methyltransferase (F: GGC ATT CGA CTT TGC CG, R: GTA ATG GCA CTC GAG GAA C), and *CLASSY1* (F: AAA TGA CTA CAA GGA CAG ACC, R: GGT GAG GAA GCA GCT TT)*.* The primers were designed with Primer Express software for RT-qPCR (version 3.0; ABI, Foster City, CA, USA). The PCR conditions used were 95°C for 10 min, then 60 cycles of 95°C for 15 s, 60°C for 30 s, 72°C for 20 s followed by cooling. The relative quantification values were obtained by using LightCycler^®^ 480 Software (version 1.5.1.62; ROCHE). Data were analyzed with the R V3.9.0 (Agricolae and dplyr packages; Team, 2015) by using logarithmic normalization transformations, then performing a multiple-factor ANOVA, followed by a honestly significant difference (HSD)–Tukey’s pairwise comparison test.

*Small RNA-seq and RNA-seq.* Whole*-*leaf (No. of *clsy1* M1-559 = 3 per treatment and No. of WT/Kitaake = 3 per control and flooded treatments) samples were ground using a ball-mill tissue grinder (Genogrinder 2000; SpexCentriprep Inc., Metuchen, NJ, USA) for 15 s at 2,000 strokes/min under liquid nitrogen conditions. RNA was extracted from the samples using Zymo^®^ following manufacturer instructions. The RNA concentration was measured using QUBIT^TM^ RNA HS assay kit (ThermoFisher Scientific) following the manufacturer instructions and RNA quality was measured using a Nanodrop (ThermoFisher Scientific). QuantSeq 3′-mRNA-seq library (Lexogen, Vienna, Austria) preparation kit and Small RNA-seq library (Lexogene, Vienna, Austria) preparation kit were used following the manufacturer instructions. Libraries were sequenced using NovaSeq6000 by multiplexing into one lane all the RNA-seq or small RNA-seq libraries at Duke University genomics core facility.

*Bisulfate sequencing.* Whole-leaf tissue (No. of FN-559-S = 3 per treatment and No. of WT/Kitaake = 3 per control and flooded treatments) samples were ground using a ball-mill tissue grinder (Genogrinder 2000; SpexCentriprep Inc., Metuchen, NJ, USA) for 15 s at 2,000 strokes/min under liquid nitrogen conditions. DNA was extracted using the GeneJET plant genomic DNA kit (ThermoFisher Scientific) following the manufacturer instructions. The DNA quality was measured using a Nanodrop (ThermoFisher Scientific) and DNA concentration was measured using QUBIT^TM^ DNA BR assay kit (ThermoFisher Scientific). DNA samples were sent for quality control, library preparation, and sequencing to GENEWIZ. Libraries were generated using swift accel-NGS methyl-seq DNA library Kit (Swift Biosciences) following the manufacturer’s protocol. Raw fastq reads were trimmed using the bbduk program from bbmap package (https://sourceforge.net/projects/bbmap/) to remove adapters and 10 bp from the end of reads as suggested by the manufacturer of the library prep kits (Settings used: *k* = 30, mink = 5, hdist = 2, hdist2 = 1). Trimmed reads were then aligned to the reference genome IRGSP1.0 using bismark 0.21 (https://www.bioinformatics.babraham.ac.uk/projects/bismark/). Alignments were then deduplicated and methylation information was extracted using bismark 0.21 with default parameters and a –no_overlap switch. The CpG methylation information was then used for downstream differential methylation analysis using methylKit 1.8.1 default parameters (http://bioconductor.org/packages/release/bioc/html/methylKit.html), methylation sites with minimal coverage of eight were retained and used for downstream analysis. Differentially methylated sites kept for further analysis had a minimum of 25% differential methylation and the *P*-value corrected for false-discovery rate using sliding linear model (SLIM; *P*-values to *q*-values; Wang et al., 2010) was ≤0.05. Differential methylation analyses were annotated using genomation 1.14.0 default parameters (http://bioconductor.org/packages/release/bioc/html/genomation.html) with data from EnsemblPlants release 44. Differential methylation was calculated by subtracting control from treatment, with the mutant used as control and the WT as treatment. The Integrated Genome Browser (Freese et al., 2016) was used to generate data visualization using the RAP-DB genome annotation (Kawahara et al., 2013; Sakai et al., 2013).

*Data analysis of bisulfite sequencing data.* CpG methylation calls (deduplicated.bismark.cov.gz file of each sample) were used as the input for the analysis to generate all the graphs. Data were filtered with minimum coverage ≥8, normalized among samples, then merged together. The results were further filtered with a *q*-value <0.05 and differential methylation level ≥25%. R package methylKit 1.8.1 with default parameters (http://bioconductor.org/packages/release/bioc/html/methylKit.html) was used for downstream differential methylation analysis. R package genomation 1.14.0 with default parameters (http://bioconductor.org/packages/release/bioc/html/genomation.html) was used for annotation. Depending on the sample size for each set it will either use Fisher’s exact or logistic regression to calculate *P*-values. *P*-values were adjusted to *q*-values using the SLIM method (Wang et al., 2010). If there were replicates, the function automatically used logistic regression. Using the significantly DML of *clsy1* (M1-559) flooded versus WT flooded comparison, we performed pathway analysis using gramene.org.

*Small RNA-seq data analysis.* Raw fastq reads were analyzed using the sRNA_snakemake_workflow (https://github.com/boseHere/sRNA_snakemake_workflow). Using default parameters, raw reads were trimmed using trimgalore, followed by filtering out noncoding RNAs and chloroplast/mitochondrial reads using bowtie (IRGSP-1.0). Reads were aligned to the reference genome (IRGSP-1.0) using Shortrack (Axtell, 2013; Johnson et al., 2016) using the sRNA_snakemake_workflow parameters. The result file was divided by sample using samtools splits flag, mapped reads were extracted using samtools view, converted to fastq using samtools bam2fq, retrieving quality coding for fastq from the filtered-out plastid reads. Finally, we produced length profiles and fastqc reports. RPKM reads results were analyzed using DESeq (https://bioconductor.org/packages/release/bioc/html/DESeq.html; Anders and Huber, 2010) using an additive model (log10(rpkm + 1)∼genotype+treatment). Normalized RPKMs from DESeq analysis were used for regularized discriminant analysis (RDA; Legendre and Legendre, 1998) in R to determine if the RPKM covaried with the treatment or genotype variables. Using the count data, we selected only 24-nt dice call clusters to run the DEseq additive model on normalized count data (∼ genotype + treatment).

*RNA-seq data analysis.* Raw fastq reads were analyzed using bluebee FWD Rice (IRGSP-1.0) Lexogen QuantSeq 2.2.3. Parameter settings were as follows: Trimming: bbduk v35.92 (Settings used: k = 13, ktrim=r, useshortkmers=t, mink = 5, qtrim=r, trimq = 10, minlength = 20); Read QC: FastQC v0.11 (Settings used: -t 8 –nogroup); Alignment: STAR v2.5.2a (Settings used: -runThreadN 8, -outFilterType, BySJout, -outFilterMultimapNmax 20, -alignSJoverhangMin 8, -alignSJDBoverhangMin 1, -outFilterMismatchNmax 999, -outFilterMismatchNoverLmax 0.6, -alignIntronMin 20, -alignIntronMax 1000000, -alignMatesGapMax 1000000, -outSAMattributes NH HI NM MD, -outSAMtype BAM SortedByCoordinate); Read Indexing: samtools index v1.3; Gene Read Counting: HTSeq-count v0.6.0 (Settings used: -m intersection, -nonempty, -s yes, -f bam, -r pos; Mapping QC: RSeQC v2.6.4. We ran the DEseq additive model (∼ genotype + treatment) on count data normalized by using size factors and dispersion parameters with default settings. These transformations take into consideration the variance of the observed count data and its mean value (Love et al., 2014). Gene expression differences were estimated using DESeq default settings; this program performed for each gene a hypothesis test to determine whether evidence is sufficient to decide against the null hypothesis. For further analysis, we used the adjusted *P*-value to select genes of interest (Love et al., 2014). Genes differentially expressed by genotype contrast were used for pathway analysis using gramene.org.

### Accession numbers

Sequence data for BGIOSGA026441 from *O. japonica* reference MSU_osa1r7 can be found using the accession numbers XM_015789529.1/LOC9266851 (Os07g0692401; methyltransferase), and XM_015790931.1/LOC4344376 (Os07g0692600; CLASSY1).

## Supplemental data

The following materials are available in the online version of this article.

**Supplemental Figure S1.** Distribution of germination phenotype in flooded (AG) and control environments at 21 DAS.

**Supplemental Figure S2.** Meta-analysis results performed on the MAGMA gene-to-SNP analyses from the wet and dry seasons.

**Supplemental Figure S3.** Phenotype of the mutant lines and WT under flooding conditions.

**Supplemental Figure S4.** Profiles of methylation and gene expression for the two genes in the seed development pathway.

**Supplemental Figure S5.** RPKM profiles of siRNA in leaves from WT and FN-559-S under control and flooded treatments.

**Supplemental Figure S6.** Profiles of methylation and gene expression within the region of interest in chromosome 7.

**Supplemental Material S1.** Information on the linear and RDA models including ANOVA.

**Supplemental Material S2.** Translation of coding region from WT Kittake and FN-559-S.

**Supplemental Table S1.** Phenotypic data for all rice lines in wet and dry seasons.

**Supplemental Table S2.** Phenotypic data for indica subpopulation in wet (square root of phenotype) and dry (phenotype) seasons including seed age for wet season.

**Supplemental Table S3.** Gene ontology enrichment results determined using AgriGO from meta-analysis that had a permuted value ≤0.01.

**Supplemental Table S4.** Methylation statistics per sample.

**Supplemental Table S5.** Percentage of DML overlapping with genome features.

**Supplemental Table S6.** Significantly DML using CpG methylation events.

**Supplemental Table S7.** Metabolic pathway analysis (gramene.org) of the 395 DML.

**Supplemental Table S8.** Cluster-specific differential expression of 24nt-siRNA between CLSY1 mutant and WT under control and flooded conditions.

**Supplemental Table S9.** Expression levels of genes that show significant differences with an adjusted *P*-value = <0.05.

## Supplementary Material

kiab100_Supplementary_Data

## Notes

L.C.-D. Designed the project, performed experiments, analyzed data and wrote the article with contributions of all authors; S.G. Designed the project, performed field experiments; F.A.Q. Performed field experiments, analyzed data and edited the manuscript; T.M.-O. Designed the project, supervised experimental design, supervised and performed data analysis and edited the manuscript; S.D. Designed the project, supervised experimental design, supervised data analysis and edited the manuscript.

The author responsible for distribution of materials integral to the findings presented in this article in accordance with the policy described in the Instructions for Author (https://academic.oup.com/plphys/pages/general-instructions) is: Lina Castano-Duque (Lina.Castano.Duque@usda.gov)
