# Supplementary material for: An epigenetic pathway in rice connects genetic variation to anaerobic germination and seedling establishment
Source: Plant Physiol. 2021 Feb 26;186(2):1042–59. doi: 10.1093/plphys/kiab100 (PMC8195528; doi:10.1093/plphys/kiab100)
Supplement: kiab100_Supplementary_Data [file kiab100_supplementary_data.zip › pp.01259.2020-s02.pdf]

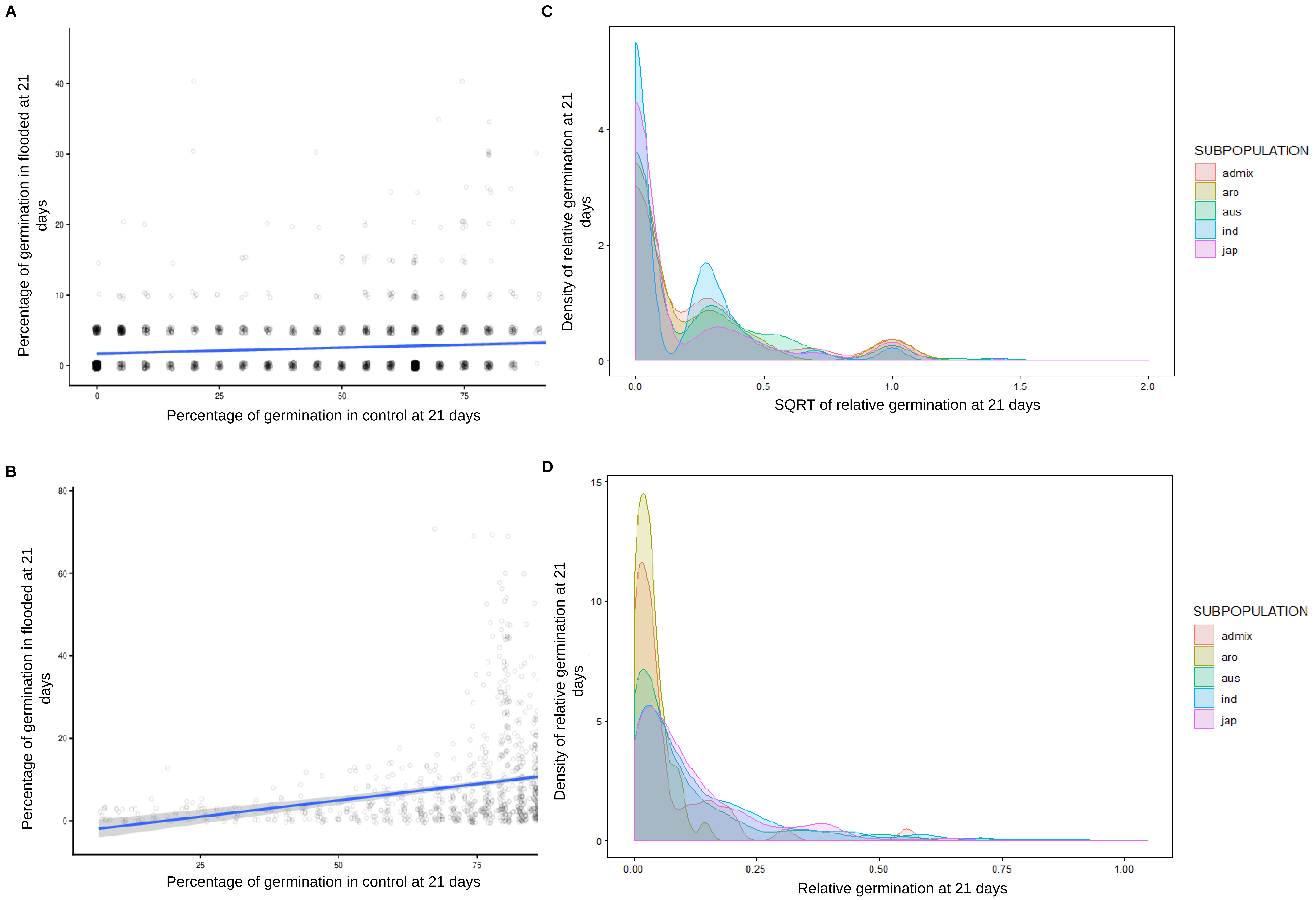

Supplemental Figure 1 Distribution of germination phenotype in flooded (AG) and control environments at 21 DAS (a) percentage of germination distribution in wet and (b) dry seasons. c) Density of relative germination, defined as the ratio of percentage of germination in flooded and in control environments at twenty-one days after seeding for the wet season and (d) dry season. Rice subpopulation shown in different colors inside the bar-graph (Salmon – *admixed*, dark green – *aromatic*, light green – *aus*, blue – *indica*, fuchsia – *japonica*). In (a) and (b), data points have been jittered around their x-axis and y-axis coordinates and the opacity of the data point location indicates the density of data points in the graph area.

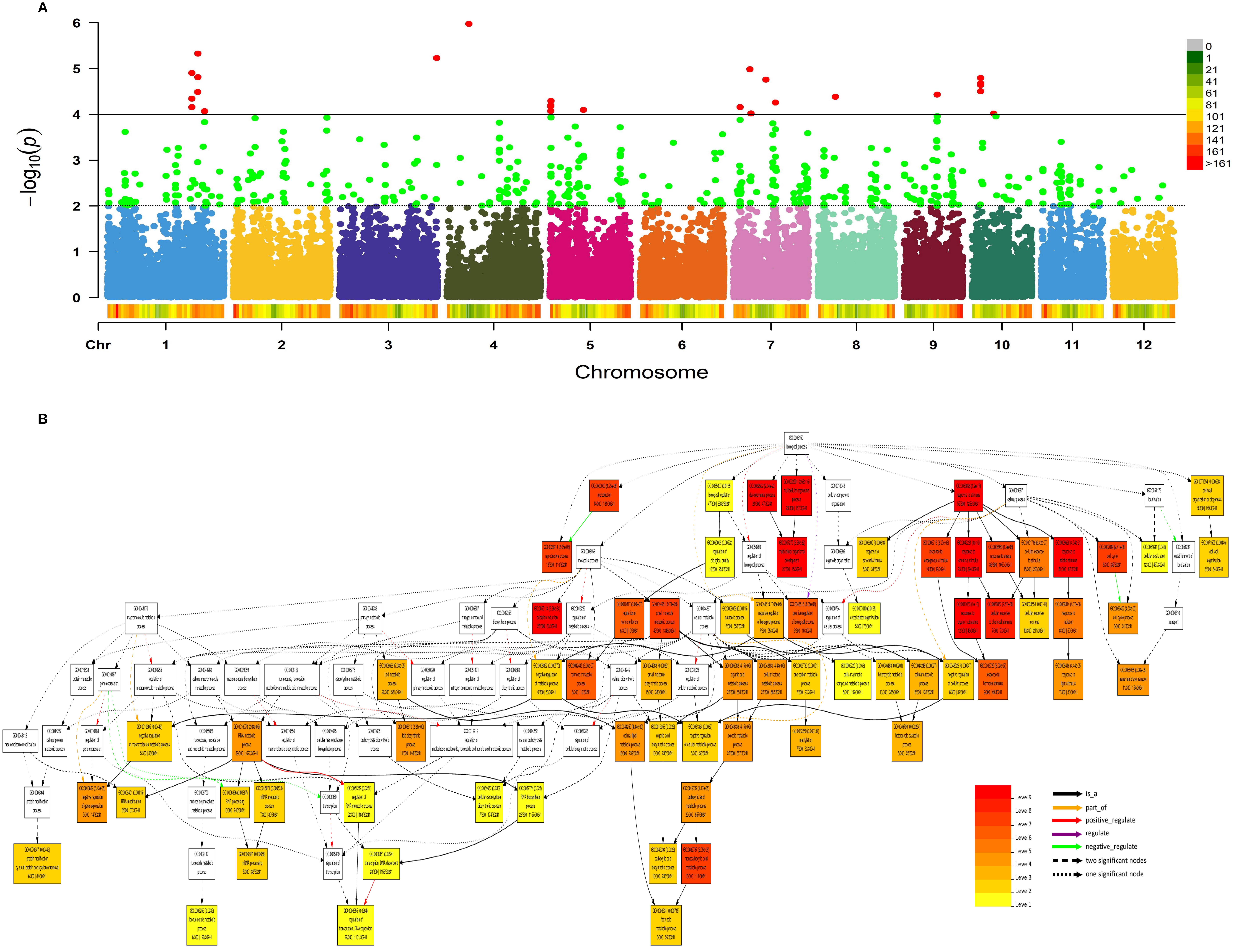

Supplemental Figure 2. Meta-analysis results performed on the MAGMA gene-to-SNP analyses from the wet and dry seasons. a) Manhattan plot of MAGMA-meta analysis, and b) gene ontology analysis of biological process using the MAGMA meta-analysis genes with adjusted p-value ≤ 0.01. In Manhattan plot each dot is a gene and the horizontal lines are the threshold for significant –log(p-value*). Bold line: 4 (p-value ≤ 0.0001)) and dashed line: 2 (p-value ≤ 0.01). SNPs within a chromosome are colored as follows: chromosome 1-blue, 2-yellow, 3-purple, 4-olive green, 5-hot pink, 6-orange, 7-light pink, 8-blue green, 9-maroon, 10-green, 11-blue, and 12-yellow. Heat maps under each chromosome represent the density of genes in a genomic region (see key to heat map in upper right corner). Gene ontology annotations were performed using AgriGO online tools. In boxes: colors represent statistical significance: darker and redder colors indicate greater statistical significance. The numbers in parentheses are the enrichment multiple-test adjusted p-values; first ratio is the number of GO terms of proteins that were searched from MAGMA meta-analysis genes and background (Rice TIGR gene model)*;* the second ratio is the total number proteins queued and background (Rice TIGR gene model). *P-values were corrected for multiple testing permutation in MAGMA.

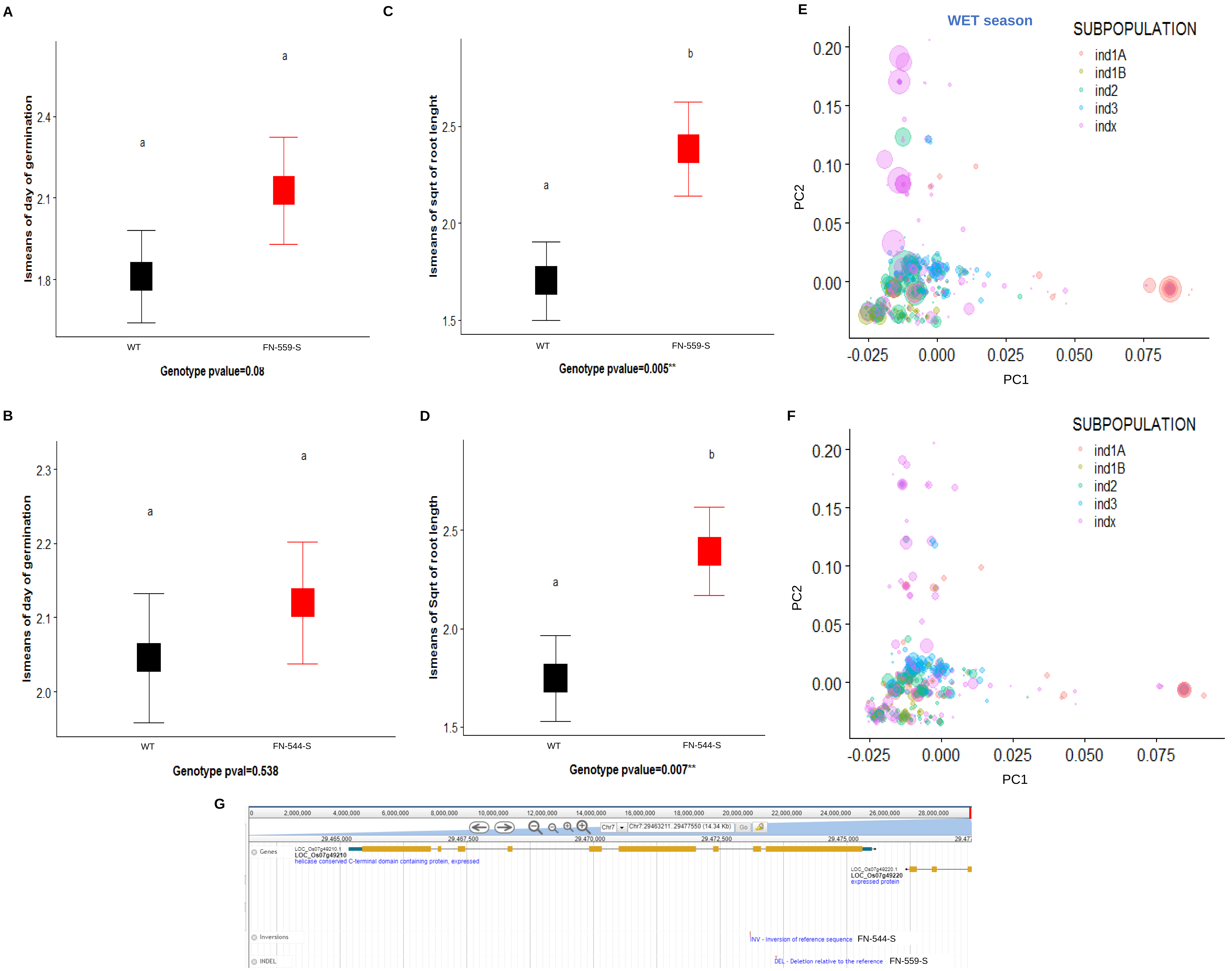

Supplemental Figure 3. Phenotype of the mutant lines and wild type under flooding conditions. a) Day of germination (square root transform of least-square means) of the FN-559-S (red) and wild type (black) at day 14 in flooding environment. b) Square root of the least-square means of day of germination of FN-544-S (red) and wild type (black) at day 14 in flooding environment. c) Square root of the root length of FN-559-S (red) and wild type (black) at day 14 in flooding environment. d) Square root of the root length of FN-544-S (red) and wild type (black) at day 14 in flooding environment. FN-559-S and FN-544-S have red color filling in plots and wild type has black color filling in plots. In x-axis is the p-value of one-way ANOVA (FN-559-S and FN-544-S; Letters on top of square plots indicate the HSD-Tukey test results with a p-value<0.05; error bars represent standard error). E and F show anerobic germination phenotype and SNP PCA. Two dimensional PCA of SNP composition of the region of interest in chromosome 7 from the 693,502 SNP genotype data for e) wet and f) dry seasons with phenotype data. Phenotype data is relative anaerobic germination, represented by diameter of the circles. Color of the circles represents the divisions of the *indica* subpopulation, salmon: *ind1a*, light-green: *ind1B*, dark-green: *ind2*, blue: *ind3* and fuchsia: *indx*. Figs. 3a and 3b show the same accessions in the same arrangement (based on genome-wide PCA), but circle sizes differ because trait expression differs between the wet and dry environments. g) Genomic location of the FN-559-S and FN-544-S mutants.

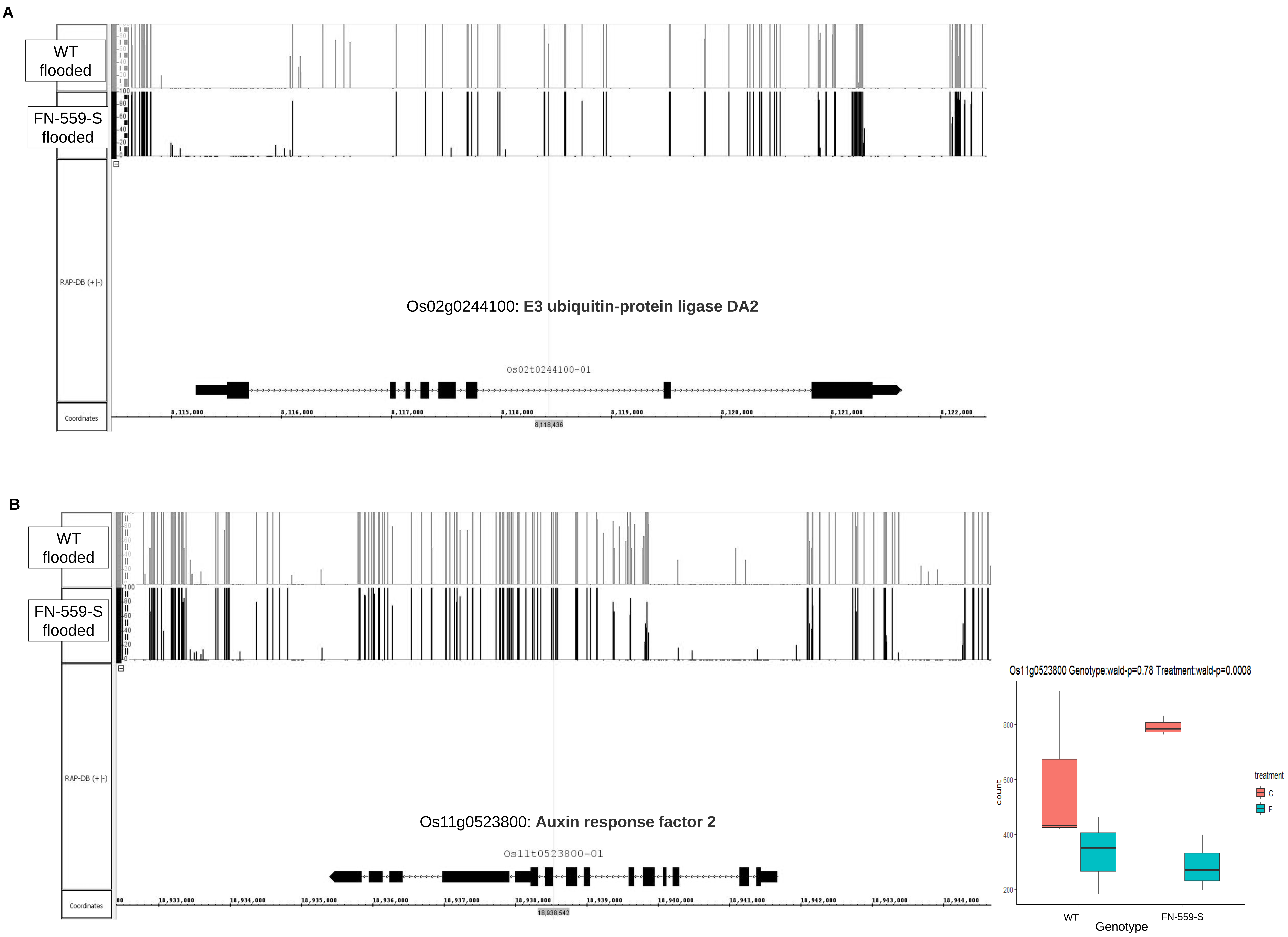

Supplemental Figure 4. Profiles of methylation and gene expression for the two genes in the seed development pathway a) Methylation profiles of DML in leaves of FN-559-S (red bars) and WT (green bars) under flooded environment for Os02g0244100: E3 ubiquitin-protein ligase DA2. b) Methylation profiles of DML in leaves of FN-559-S (black bars) and WT (grey bars) under flooded environment and gene expression profiles in leaves of wild type and FN-559-S under flooded and control environment. Genome annotation is RAP-DB 2011 version. Counts from transformed count data from additive model Os11g0523800: Auxin response factor 2. Error bars show the standard error and blue color indicates flooded treatment and salmon color indicates control treatment. Wald-p-value was calculated using DESeq additive model of RNA count levels.

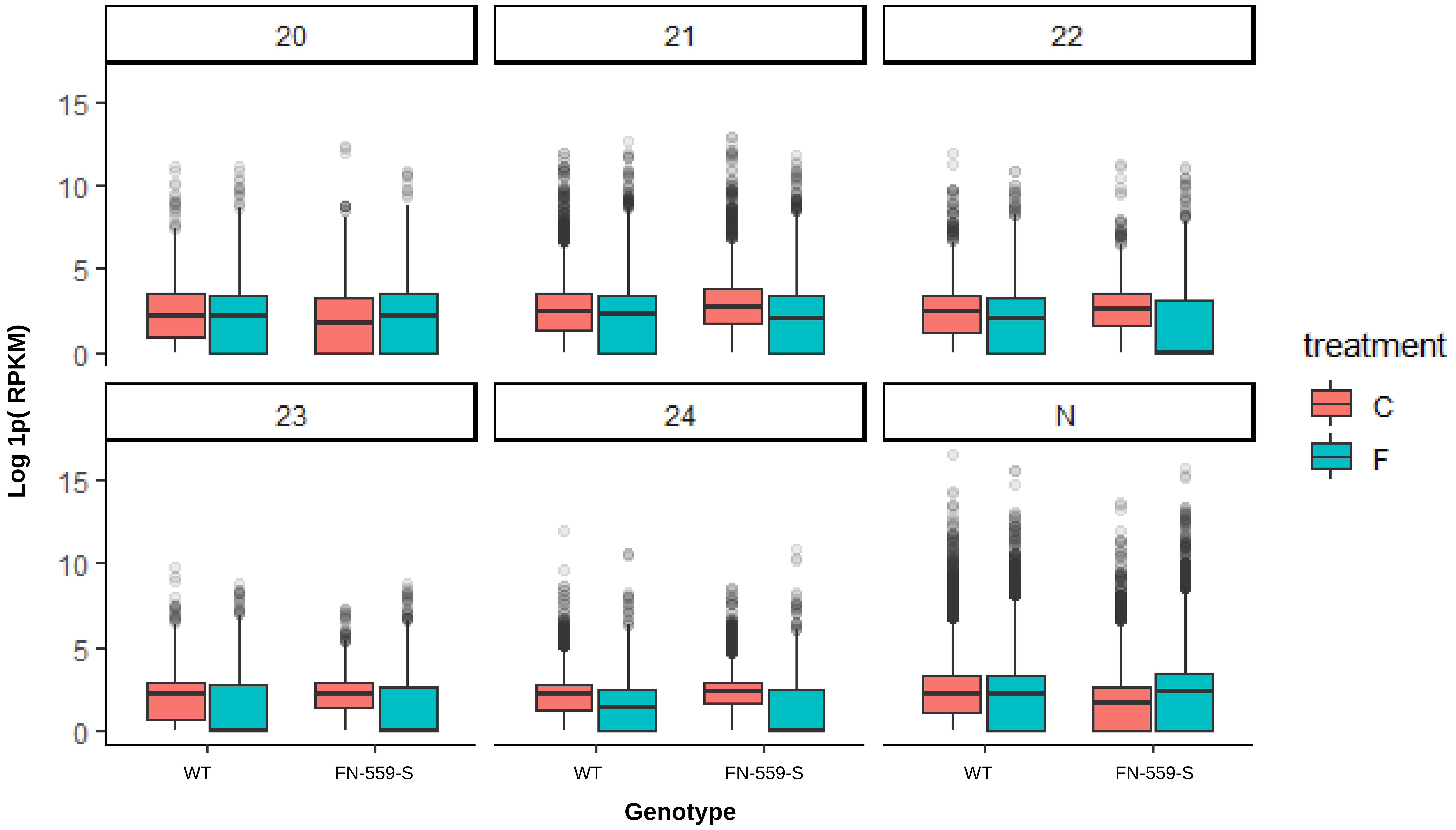

Supplemental Figure 5. RPKM profiles of siRNA in leaves from wild type and FN-559-S under control and flooded treatments. RPKM were normalized by using DEseq dispersion coefficients and size factor normalization. FN-559-S siRNA profiles are different from wild type. Figure faceted by nucleotide length of siRNAs, based on dicercall size generated using Shortstack, error bars show the standard error and blue color indicates flooded treatment and salmon color indicates control treatment.

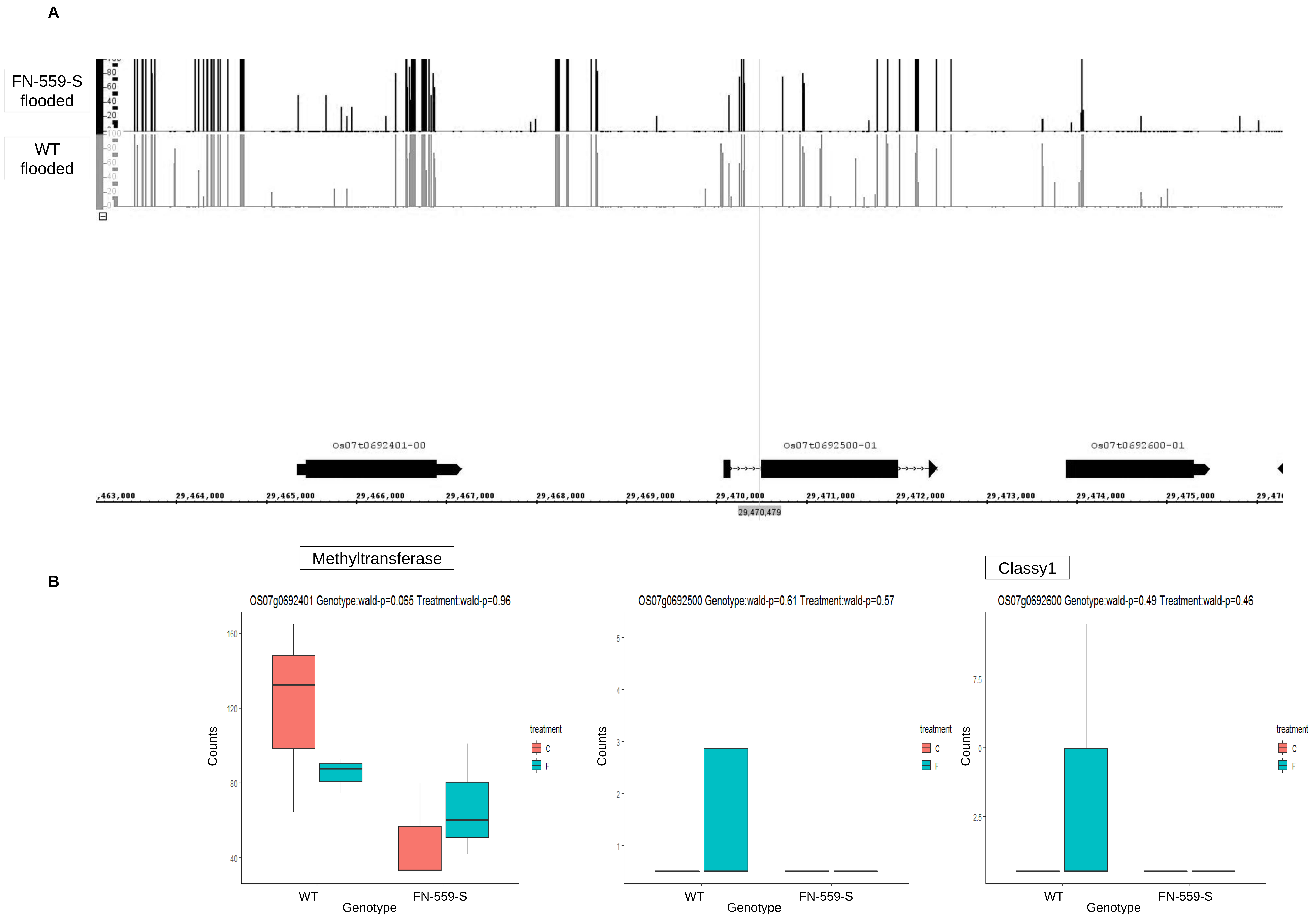

Supplemental Figure 6. Profiles of methylation and gene expression within the region of interest in chromosome 7. a) Methylation profiles of DML in leaves of FN-559-S (black) and WT (grey) under flooded environment. b) Gene expression profiles in leaves of wild type and FN-559-S under flooded and control environment. Genome annotation is RAP-DB 2011 version. Counts are from transformed count data from additive model for Os07g0692401 (methyltransferase), Os07g0692500 (Unknown gene) and Os07g0692600 (CLASSY1). Error bars show the standard error. Blue indicates flooded treatment and salmon indicates control treatment. Normalized counts for RNA-expression were calculated after performing dispersion coefficient and size factor calculations per library using DESeq. Wald p-value was calculated using DESeq additive model of RNA count levels.

Supplemental material 1. Information on the linear and RDA models including ANOVA

Try the additive model using only leave tissues rpkm data after doing normalization with DEseq taking into account the library size and dispersion factors. Then used the normalized values to run an RDA model of the RPKM (Multivariate). Significant differences between treatments only

Call: rda(formula = log1p(rpkm) ~ treatment + genotype, data = phenotype, estimate.error =
TRUE) Inertia Proportion Rank
Total 3.818e+04 1.000e+00
Constrained 1.144e+04 2.996e-01 2
Unconstrained 2.674e+04 7.004e-01 9

Inertia is variance
Eigenvalues for constrained axes:
RDA1 RDA2
8429 3008

Eigenvalues for unconstrained axes:
PC1 PC2 PC3 PC4 PC5 PC6 PC7 PC8 PC9
5968 3703 3636 3606 3521 2628 1792 1078 808

r.squared 0.2995822
adj.r.squared 0.1439338

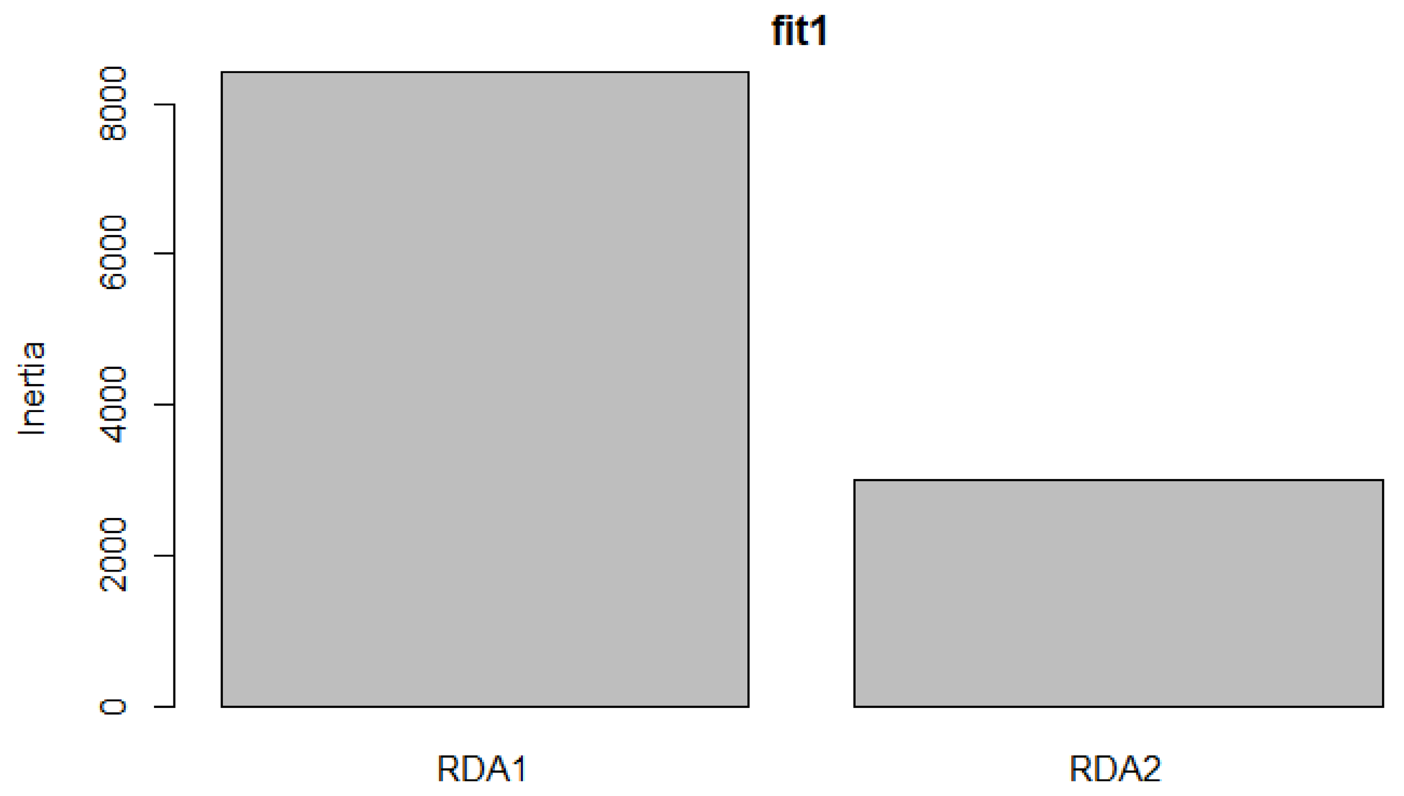

Permutation test for rda under reduced model
Marginal effects of terms
Permutation: free
Number of permutations: 999
Model: rda(formula = log1p(rpkm) ~ treatment + genotype, data = phenotype, estimate.error =
TRUE)
Df Variance F Pr(>F)
treatment 1 8397.9 2.8265 0.020 *
genotype 1 3039.4 1.0230 0.234
Residual 9 26740.3
--- Signif. codes: 0 ‘***’ 0.001 ‘**’ 0.01 ‘*’ 0.05 ‘.’ 0.1 ‘ ’ 1

Supplemental material 2. Translation of coding region from wild type Kittake and FN-559-S. In CDS box (Chr7:29473405..29475312 (+ strand) class=CDS length=1908) the 11 base pair deletion is highlighted in grey. In wild type and FN-559-S amino acid sequence boxes the start codon (Met) is highlighted in green, and stop is highlighted in red. For reference the amino acid sequence prior to the deletion is highlighted in blue.

CDS > Chr7:29473405..29475312 (+ strand) class=CDS length=1908

ttcaatggaaatgactacaaggacagacctggatgcagcaatatttgccttgatgatgatatacttgatccttctcttcttgcaaacttggcaccagagttgtcagaattgaagaactcagggagcgtgtggagtgcaatatctgatcttgatccaaagctgcttcctc
acc**agagaaaagca**ctagattttctgtggaaaaatttggcggggtcgatacaggttgaaggaatggataactcaaatgtcagtacaggtggctgtgtgatagcacacactcctggatctgggaaaacactgctgcttatctcatttctggtgagttacatgaaag
ctcacccaagaagccggccattggtccttactccaaaagctgcaattcatacatggaagagagaatttgagaaatggggcatttcacttcctttacatgtgttccaccatgctaacagaagtggcaagccgttgggggcaatggattctaaactgcggtcgttatta
aataattttcacagaccaacctggacaaatatgcgccttatggattctttggacaaactattcaagtggcatgcacatccaagcgtcctcctcatgacatactcatctttcttagggatgacgaaacaagactcaaaagtgcgaaaccgataccgagaatttatcg
cagaggtcctgatgaacaaccctggactcttgattcttgacgagggcacaacccaagaagcaacaagtccaagttgaggaagctgctgatgaaggtgaagactgagttcagaatcctcttatctggcacggccttccagaacaactttgaagagtacttcaa
caccctatgtttggccagacctcggtttatcggtgatatcatgagtgaactagtgccagagaggaaaagagaaacagttggcaggagagccaaacatcaagaagcagtggcacgccgcgcctttgtggagaaagtgggccagaagattgagtctgacaac
aaacatatcagaagtgatggtatcagtttgctaaacaagcttactcgtggattcatagattcattcgagggagcgaagctgatcaaccttccagggatacatgtgtataccgtgttcatgaagcccacggacattcaggaggagatgctggcaaaggtaacaat
gcccaagctcgggtcgtcccggttccctctagaggttgagctgctcatcacgattggctccatccatccttggctcataaaaacgacgaaggccgtcagcaccttcttcagtccagccgaagtgaagaaggttgagaggtacaagcgagacttcgcggcggg
gtgcaaggccaaatttgtgatcgatctgctgcacaagtcgtcgttcagaggggagagggtgctgatattctgccacaacgtgtccccgatcacgttcctggtgaagctgatagagatggtgtttgggtggcggctcggggaggaggtgctggtgcttcagggtga
tcaggagctgcctgtccggtccgatgtgatggacaagttcaacggcgacagcgcggggaagaggaaggtgctgatcgcgtcgacaacggcatgcgcggaggggatcagcttgacaggcgcgtcgaggctggtgatgctggactcggagtggaaccact
ccaagacgaggcaggcgatcgcgcgggcgttccggcgtgggcaggagaggacggtgtacgtctacctcctggtggcatctgggacatgggaggaggagaagtacaacagcaacaggaggaaagcttggatgtccaaaatggtgttcctgggacgctat
gttgatgattcctcgcaaaaccgtgtcactgacatcgatgatgaggtcttgaaggagcttgccgatgaagatcacaccggcaccttccatatgattgtcaagcaagactga

5'3' Frame 1 Wild type

F N G N D Y K D R P G C S N I C L D D D I L D P S L L A N L A P E L S E L K N S G S V W S A I S D L D P K L L P H Q R K A L D F
L W K N L A G S I Q V E G Met D N S N V S T G G C V I A H T P G S G K T L L L I S F L V S Y Met K A H P R S R P L V L T P K A A I
H T W K R E F E K W G I S L P L H V F H H A N R S G K P L G A Met D S K L R S L L N N F H R P T W T N Met R L Met D S L D K L
F K W H A H P S V L L Met T Y S S F L G Met T K Q D S K V R N R Y R E F I A E V L Met N N P G L L I L D E G H N P R S N K S K
L R K L L Met K V K T E F R I L L S G T A F Q N N F E E Y F N T L C L A R P R F I G D I Met S E L V P E R K R E T V G R R A K H Q
E A V A R R A F V E K V G Q K I E S D N K H I R S D G I S L L N K L T R G F I D S F E G A K L I N L P G I H V Y T V F Met K P T D I
Q E E Met L A K V T Met P K L G S S R F P L E V E L L I T I G S I H P W L I K T T K A V S T F F S P A E V K K V E R Y K R D F A A
G C K A K F V I D L L H K S S F R G E R V L I F C H N V S P I T F L V K L I E Met V F G W R L G E E V L V L Q G D Q E L P V R S
D V Met D K F N G D S A G K R K V L I A S T T A C A E G I S L T G A S R L V Met L D S E W N H S K T R Q A I A R A F R R G Q E R
T V Y V Y L L V A S G T W E E E K Y N S N R R K A W Met S K Met V F L G R Y V D D S S Q N R V T D I D D E V L K E L A D E D H
T G T F H Met I V K Q D Stop

5'3' Frame 1 FN-559-S

F N G N D Y K D R P G C S N I C L D D D I L D P S L L A N L A P E L S E L K N S G S V W S A I S D L D P K L L P H P R F S V E K
F G G V D T G Stop R N G Stop L K C Q Y R W L C D S T H S W I W E N T A A Y L I S G E L H E S S P K K P A I G P Y S K S C N S
Y Met E E R I Stop E Met G H F T S F T C V P P C Stop Q K W Q A V G G N G F Stop T A V V I K Stop F S Q T N L D K Y A P Y G F
F G Q T I Q V A C T S K R P P H D I L I F L R D D E T R L K S A K P I P R I Y R R G P D E Q P W T L D S Stop R G A Q P K K Q Q V
Q V E E A A D E G E D Stop V Q N P L I W H G L P E Q L Stop R V L Q H P Met F G Q T S V Y R Stop Y H E Stop T S A R E E K R
N S W Q E S Q T S R S S G T P R L C G E S G P E D Stop V Stop Q Q T Y Q K Stop W Y Q F A K Q A Y S W I H R F I R G S E A D
Q P S R D T C V Y R V H E A H G H S G G D A G K G N N A Q A R V V P V P S R G Stop A A H H D W L H P S L A H K N D E G R Q
H L L Q S S R S E E G Stop E V Q A R L R G G V Q G Q I C D R S A A Q V V V Q R G E G A D I L P Q R V P D H V P G E A D R D
G V W V A A R G G G A G A S G Stop S G A A C P V R C D G Q V Q R R Q R G E E E G A D R V D N G Met R G G D Q L D R R V E
A G D A G L G V E P L Q D E A G D R A G V P A W A G E D G V R L P P G G I W D Met G G G E V Q Q Q Q E E S L D V Q N G V P
G T L C Stop Stop F L A K P C H Stop H R Stop Stop G L E G A C R Stop R S H R H L P Y D C Q A R L

Supplemental Table 4. Methylation statistics per sample. Bismark Methylation Extractor was used to call methylation status of individual bases in the deduplicated aligned reads.

Sample	Total C	methylated CpG	% methylated CpG	methylated CHG	% methylated CHG	methylated CHH	% methylated CHH	unmethylated CpG	unmethylated CHG	unmethylated CHH
1 Kitaake_Control	1.07E+09	79803930	43.3	40287127	22.2	24616660	3.5	1.04E+08	1.41E+08	6.79E+08
2 Kitaake_Control	1.06E+09	80751658	43.7	40599596	22.5	23156037	3.3	1.04E+08	1.4E+08	6.68E+08
3 Kitaake_Control	9.67E+08	71611787	42.4	36229606	22	20470674	3.2	97336113	1.29E+08	6.13E+08
4 Kitaake_Flooded	8.3E+08	60349966	41.6	30428631	21.5	15140494	2.8	84796269	1.11E+08	5.28E+08
5 Kitaake_Flooded	6.21E+08	44798077	41	22584737	21.2	11438872	2.8	64577898	83797017	3.94E+08
6 Kitaake_Flooded	7.07E+08	51031563	41.2	25686507	21.2	13403765	2.9	72812042	95240208	4.49E+08
7 M1_Control	8.02E+08	60312389	43.3	30758878	22.5	16646964	3.2	78851242	1.06E+08	5.1E+08
8 M1_Control	8.61E+08	62534284	41.5	31328117	21.3	17397366	3.1	88307882	1.16E+08	5.46E+08
9 M1_Control	7.81E+08	56354592	41.2	27856765	20.9	16014717	3.1	80552762	1.06E+08	4.95E+08
10 M1_Flooded	6.73E+08	49674828	42.7	25055852	21.9	14170979	3.2	66769679	89236686	4.28E+08
11 M1_Flooded	7.01E+08	50973596	41.6	25824889	21.6	13299175	2.9	71651405	93822160	4.46E+08
12 M1_Flooded	5.83E+08	42916696	42.9	21849771	22.1	10968428	2.9	57192240	76961096	3.73E+08

Supplemental Table 5. Percentage of differentially methylated loci (DML) overlapping with genome features. The DML results were determined by filtering loci with a p-value < 0.05 and differential methylation level ≥ 25%. The filtered results were annotated with transcription start sites (TSS) information from Refseq.

Comparison	promoter	exon	intron	intergenic
Kitaake Control vs. Kitaake Flooded	36.36%	6.82%	0%	56.82%
FN-559-S Control vs. FN-559-S Flooded	46.67%	2.22%	6.67%	44.44%
Control FN-559-S vs. Control Kitaake	45.31%	6.55%	5.76%	42.38%
Flooded FN-559-S vs Flooded Kitaake	42.22%	4.75%	6.6%	46.44%
